# Co-administration of temozolomide (TMZ) and the experimental therapeutic targeting miR-10b, profoundly affects the tumorigenic phenotype of human glioblastoma cells

**DOI:** 10.3389/fmolb.2023.1179343

**Published:** 2023-06-15

**Authors:** Ming Chen, Bryan Kim, Neil Robertson, Sujan Kumar Mondal, Zdravka Medarova, Anna Moore

**Affiliations:** ^1^ Precision Health Program, Michigan State University, East Lansing, MI, United States; ^2^ Department of Radiology, College of Human Medicine, Michigan State University, East Lansing, MI, United States; ^3^ Department of Biomedical Engineering, College of Engineering, Michigan State University, East Lansing, MI, United States; ^4^ Transcode Therapeutics Inc., Boston, MA, United States

**Keywords:** glioblastoma multiforme, microRNA, temozolomide, *in vitro* studies, nanoparticles

## Abstract

**Introduction:** Recent studies have shown that miRNA-10b is highly expressed in high-grade glioblastoma multiforme (GBM), and its inhibition leads to deregulation of multiple pathways in tumorigenesis, resulting in repression of tumor growth and increased apoptosis. Thus, we hypothesized that suppressing miR-10b could enhance the cytotoxicity of conventional GBM chemotherapy with temozolomide (TMZ).

**Methods:** Inhibition of miR-10b in glioblastoma cells was achieved using an experimental therapeutic consisting of anti-miR10b antagomirs conjugated to iron oxide nanoparticles (termed MN-anti-miR10b). The nanoparticles serve as delivery vehicles for the antagomirs as well as imaging reporters guiding the delivery in future animal studies.

**Results:** Treatment of U251 and LN229 human glioblastoma cells with MN-anti-miR10b led to inhibition of miR-10b accompanied by repression of growth and increase in apoptosis. We next explored whether MN-anti-miR10b could enhance the cytotoxic effect of TMZ. During these studies, we unexpectedly found that TMZ monotherapy increased miR-10b expression and changed the expression of corresponding miR-10b targets. This discovery led to the design of a sequence-dependent combination treatment, in which miR-10b inhibition and induction of apoptosis by MN-anti-miR10b was followed by a sub-therapeutic dose of TMZ, which caused cell cycle arrest and ultimately cell death. This combination was highly successful in significant enhancement of apoptosis and decrease in cell migration and invasiveness.

**Discussion:** Considering the unexpected effects of TMZ on miR-10b expression and possible implications on its clinical application, we reasoned that comprehensive *in vitro* studies were warranted before embarking on studies in animals. These intriguing findings serve as a solid foundation for future *in vivo* studies and offer promise for the successful treatment of GBM.

## 1 Introduction

Glioblastoma multiforme (GBM), the most common and aggressive primary brain tumor in adults, has the highest mortality rate among all brain malignancies. This is primarily due to persistent tumor growth and highly aggressive invasion, as well as resistance to chemotherapy and ionizing radiation therapy (Stupp et al., 2005).

microRNAs (miRNAs) are small, highly conserved, non-coding RNA molecules involved in regulating diverse biological processes, including cell development, differentiation, proliferation, and apoptosis. Recent studies have shown that miRNAs may help to regulate several aspects of tumorigenesis, including invasiveness, DNA repair, and acquired resistance to genotoxic cancer therapy.

One notable microRNA, miR-10b, is highly expressed in high-grade glioblastoma while absent in normal neuroglial cells of the brain (Gabriely et al., 2011; Sun et al., 2011). miR-10b targets include RhoC and uPAR whose levels are directly proportional to the level of miR-10b, causing enhancement in the invasive capabilities of high-grade glioma (Sasayama et al., 2009). The tumor suppressor HOXD10 has also been identified as a direct miR-10b target (Lin et al., 2012). Additionally, the direct targets of miR-10b related to cell growth include BCL2L11, TFAP2C, CDKN1A, and CDKN2A (Gabriely et al., 2011). A pro-apoptotic protein Bim, a BCL2 interacting mediator of cell death, has also been identified as a miR-10b target in GBM (Gabriely et al., 2011). Interestingly, our group identified BIM as miR-10b target in metastatic breast cancer as well (Yoo et al., 2015). In previous studies, inhibition of miR-10b restored the expression of these gene targets and decreased the growth of glioma cells through apoptosis and/or cell cycle arrest as well as by reducing angiogenesis (Sun et al., 2011).

Accumulating evidence indicates that inhibition of miR-10b in glioblastoma cells leads to deregulation of multiple pathways in tumorigenesis, resulting in repression of tumor growth and increased apoptosis, all of which could theoretically enhance the chemotherapeutic effects of cancer therapy. Since one of the phenotypic effects of miR-10b inhibition is induction of apoptosis, we hypothesized that suppressing miR-10b could enhance the cytotoxicity of conventional chemotherapy, such as temozolomide (TMZ).

Previously, we have successfully inhibited miR-10b in metastatic breast cancer using an experimental therapeutic consisting of anti-miR10b antagomirs conjugated to iron oxide nanoparticles (termed MN-anti-miR10b). Treatment of murine models of metastatic breast cancer with MN-anti-miR10b led to regression or elimination of established metastases (Yigit et al., 2013; Yoo et al., 2014a; Yoo et al., 2015; Yoo et al., 2017). The iron oxide nanoparticles used in this study served as delivery vehicles for the antagomirs as well as *in vivo* imaging reporters guiding the delivery.

In the current study, we aimed to explore whether miR-10b inhibition using MN-anti-miR10b could enhance the cytotoxic effect of TMZ in glioblastoma cells. During the course of our experiments, we unexpectedly discovered that treatment of U251 and LN229 human glioma cells with IC50 concentrations of TMZ led to an increase in miR-10b expression. This discovery led to the design of a sequence-dependent combination treatment, which relied on pre-treatment with MN-anti-miR10b to inhibit miR-10b expression and induce apoptosis, followed by a sub-therapeutic dose of TMZ. This combination was effective at repressing the tumorigenic phenotype of human glioblastoma cells.

Considering the unexpected effects of the combination treatment and the fact that TMZ is administered as a first-line treatment for GBM (Ortiz et al., 2021) and is the only FDA-approved therapy with a survival benefit for adult patients with glioblastoma (Thomas et al., 2014), we reasoned, that comprehensive *in vitro* studies were warranted before embarking on studies in animals. The intriguing findings presented here serve as a solid foundation for future *in vivo* studies and offer a promising alternative for the successful treatment of GBM.

## 2 Materials and methods

### 2.1 Antagomirs

The locked nucleic acid (LNA) antagomirs (anti-miR-10b), 50-ThioMC6-D/GTGTAACACGTCTATACGCCCA-30, directed against miRNA-10b and a mismatch scrambled sequence (scr-miR), 50-ThioMC6-D/CACAAATTCGGTTCTACAGGGTA-30, were synthesized by Eurogentec (Belgium). The 5′-Thiol-Modifier C6 disulfide (5′-ThioMC6) was inserted into both sequences for conjugation to magnetic nanoparticles for *in vitro* studies. The thiol modified oligonucleotides were activated by treatment with 3% TCEP (Tris(2-carboxyethyl)phosphine hydrochloride, Thermo Scientific Co., Rockford, IL), followed by purification with ammonium acetate/ethanol precipitation before conjugation to the nanoparticles, as described previously (Yigit et al., 2013; Yoo et al., 2014a).

### 2.2 MN-anti-miR10b synthesis and characterization

Aminated magnetic nanoparticles (MN) were synthesized using a protocol described in (Yigit et al., 2013; Yoo et al., 2014a). The nanoparticles were conjugated to the heterofunctional linker SPDP (N-succinimidyl 3-[2-pyridyldithio]-propionate; Pierce Biotechnology, Rockford, IL, United States) to provide a thiol reactive terminus for LNA conjugation. In brief, 10 mg of SPDP was dissolved in 500 mL anhydrous DMSO and incubated with MN. The LNA oligos (anti-miR-10b or scr-miR) were then conjugated to MN. The thiolated 5′ terminus of the oligo was activated via 3% TCEP treatment in nuclease free PBS. The LNA oligos were purified using ammonium acetate/ethanol precipitation method. After TCEP activation and purification, the oligos were resuspended in PBS with 50 mM EDTA and incubated with magnetic nanoparticles overnight. The resulting therapeutics (termed MN-anti-miR10b and MN-scr-miR) were purified using G-50 Sephadex quick spin columns (Roche Applied Science, Indianapolis, IN, United States). The quantification of LNA per MN was described previously (Yoo et al., 2014a). Characterization of MN-anti-miR10b and MN-scr-miR is shown in Supplementary Table S1. For transmission electron microscopy (TEM) the prepared nanoparticles in PBS were placed into the copper grids and air dried. The TEM images were captured using a high-resolution JEM-2200FS Field Emission Electron Microscope (JEOL) (Supplementary Figure S1) and confirmed crystalline lattice structure of the iron core similar to our previously published data (Yoo et al., 2021) and data by others (Roca et al., 2006; Hurley et al., 2015).

### 2.3 Cell culture

Human glioma U251, LN229 and T98G cells were obtained from the American Type Culture Collection (ATCC, Manassas, VA, United States) and maintained at 37°C with 5% CO_2_ in Dulbecco’s modified Eagle’s medium (DMEM) (Gibco, United States) supplemented with 10% fetal bovine serum (FBS), (Gibco, United States), 2 mM glutamine (Gibco, United States), 1% penicillin/streptomycin (Gibco, United States) GL261 murine glioma cells were obtained from Dr. Costas D. Arvanitis (Georgia Institute of Technology, Atlanta, GA) and maintained in DMEM supplemented with 10% FBS, 2 mM glutamine and 1% penicillin/streptomycin. All cells were seeded in 75 cm^2^ flasks and incubated at 37°C in a humidified atmosphere with 5% CO_2_.

### 2.4 IC50 determination

To determine IC50 concentrations of TMZ and MN-anti-miR10b, U251, LN229 and T98G cells (4 × 10^3^ cells/well) were seeded in 96-well plates and allowed to attach overnight. TMZ or MN-anti-miR10b were added to the wells at different concentrations (*n* = 8 wells per group/treatment) and incubated for 48 h. Cell viability was determined by the MTS [3-(4,5-dimethylthiazol-2-yl)-5-(3-carboxymethoxyphenyl)-2-(4-sulfophenyl)-2H-tetrazolium] assay according to the manufacturer’s instructions (Promega). IC50 values were calculated by nonlinear regression analysis as percent inhibition versus log inhibitor concentration. Once IC50 values were determined, they were used for combination treatment in migration, invasion, qRT-PCR and Western blotting assays.

### 2.5 Cell treatment

To evaluate the effect of the combination treatment with TMZ and MN-anti-miR10b, we treated U251, LN229, T98G and GL261 cells with IC50 concentrations of the drugs alone or in combination (TMZ concentration: 203 μM for U251; 666 μM for LN229, 500 μM for T98G and 2.58 mM for GL261; MN-anti-miR10b concentration: 0.02 μM antagomir for U251, 0.07 μM antagomir for LN229 and 0.05 μM antagomir for T98G) for 48 h. Cell viability was determined by the MTS assay as described above. The assay using increasing concentrations of unconjugated iron oxide nanoparticles or nanoparticles conjugated to the scrambled oligos was used to demonstrate the absence of nanoparticle toxicity.

### 2.6 Fluorescence microscopy

Fluorescence microscopy confirming accumulation of the therapeutic in the cells was performed after incubation with MN-anti-miR10b for 2 h in Cy5.5 and DAPI channels using Nikon Eclipse 50i fluorescence microscope with Spot Imaging 5.2 software.

To assess the expression of epithelial–to–mesenchymal transition (EMT) markers after 2 h incubation with MN-anti-miR10b, MN-scr-miR or PBS cells were fixed with 4% PFA (Electron Microscope Sciences, 15713S) in DPBS for 10 min at RT followed by permeabilization with 0.1% Triton X-100 (Sigma-Aldrich, CAS# 9036-19-5) in DPBS for 10 min at RT. Cells were then blocked with 2% BSA in DPBS for 60 min at RT. For Twist, cells were stained with mouse monoclonal IgG Twist1 (1:200, Invitrogen, Catalog # MA5-38652) primary antibodies in 0.1% BSA overnight at 4°C. Cells were then washed for 5 min with DPBS three times, then stained with secondary antibodies Alexa Fluor® 594 Goat anti-mouse IgG1 (1:300, Invitrogen, Catalog #A-21125) in 0.1% BSA for 1 h at RT. For fibronectin, cells were stained with mouse anti-human Fibronectin Alexa Fluor® 488 (1/100 dilution, Invitrogen Catalog # 53-9869-82). Cells were washed three times for 10 min with DPBS and mounted with VECTASHIELD Antifade Mounting Medium with DAPI (Vector Lab, H-1200). Slides were imaged with a fluorescence microscope as indicated above. Fiji ImageJ software (Schindelin et al., 2012) was used to analyze fluorescence intensity of images. Relative fluorescence intensity (RFI) was calculated by fluorescence intensity normalized to DAPI signals.

### 2.7 Migration and invasion assays

To establish whether MN-anti-miR10b alone or in combination with TMZ affected invasion and migration of tumor cells, we performed corresponding assays according to the manufacturer’s instructions (Cell Biolabs, San Diego, CA, United States). Briefly, cells (0.5 × 10^6^) were plated in transwell migration/invasion plates (both pore size 8 mm) and corresponding drugs were added with or without 10% FBS (fetal bovine serum) at their corresponding IC50 concentrations. The cells were incubated as specified by the manufacturer. The inserts were stained, and the membranes were imaged using light microscopy. The absorbance was read at 560 nm using a SpectraMax iD5 microplate reader (Molecular Devices, Sunnyvale, CA, United States).

### 2.8 RNA extraction and real-time quantitative RT-PCR

Total RNA from all cell lines for all treatments was purified using an miRNeasy Mini Kit (Qiagen)/RNeasy Mini Kit (Qiagen) and quantified by spectrophotometry (Nanodrop). cDNA was synthesized using miScript II RT Kit (Qiagen). Target miRNA/mRNA was amplified and measured using Bio-Rad CFX96 Real Time System. The protocol was performed for 40 cycles, comprising 95°C for 15 min, 94°C for 15 s, 57°C for 30 s. Gene expression was determined using the SYBR Select Master Mix (Applied Biosystems). U6/18S ribosomal RNA (18S rRNA) expression levels were used as the quantitative internal control. For precise quantification, the miRNA/mRNA expression level of each sample was normalized using the expression of the U6 for miRNAs or 18S rRNA for mRNAs. Data were shown as fold change (2^−ΔΔCt^).

### 2.9 SDS-PAGE and Western blotting

Cells treated with various drug combinations were washed with cold PBS and lysed in RIPA buffer containing 1 mg/mL DNAse (Roche Life Science) and supplemented with protease inhibitor cocktail (Roche Life Science). Cell lysates stored frozen at −80°C, were resolved on 4%–20% SDS-PAGE gels and transferred onto a nitrocellulose membrane. The membranes were first stained with anti-BIM (Cell Signaling Technology), anti-HOXD10 (Santa Cruz Biotechnology) or anti-PTEN (Santa Cruz Biotechnology) antibody followed by peroxidase-conjugated anti-rabbit antibody. DAB substrate (Roche Life Science) was used to develop blots. After documenting signal, the blot was subjected to a second round of immunostaining using an anti-actin HRP antibody (Santa Cruz Biotechnology).

### 2.10 Optimization of the combination treatment

To optimize the combination treatment, we evaluated the effect of simultaneous or sequential incubation with sub-therapeutic dose of TMZ in combination with MN-anti-miR10b on U251 and LN229 cells. For simultaneous treatment, MN-anti-miR10b or MN-scr-miR alone or in combination with TMZ were added to the cells and incubated for 48 h. For sequential treatment, cells were incubated first with either MN-anti-miR10b or TMZ for 24 h followed by 24 h incubation with either TMZ or MN-anti-miR10b. Induction of apoptosis was determined using Annexin V Apoptosis Detection Kit according to the manufacturer’s protocol (BioLegend, San Diego, CA). Each experiment was performed in triplicate.

In this study we used the IC15 concentration of TMZ and the IC90 concentration of MN-anti-miR10b (for U251: 36 μM TMZ and 0.18 μM antagomir; for LN229: 118 μM TMZ and 0.63 μM antagomir). Cells were not washed between the treatments.

### 2.11 Cell cycle analysis

For cell cycle analysis, cells were incubated with MN-anti-miR10b or MN-scr-miR alone or in combination with TMZ at concentrations described above for 48 h. Next, cells were washed with PBS, collected by trypsinization, and fixed in 70% ethanol precooled to −20°C. The cells were then washed twice with PBS and treated with RNase A (Thermo Fisher Scientific, Waltham, MA) for 30 min at room temperature before adding propidium iodide. Cells were analyzed on an Accuri C6 flow cytometer (BD Biosciences, San Jose, CA) and data were quantified by the FCS Express 7 (*De Novo* software, Pasadena, CA) or Flowjo 10 (BD Biosciences, San Jose, CA) software. Cell fragments comprising a sub-G1 population were gated out and not used in the analysis.

### 2.12 Statistical analysis

Statistical analysis was performed using GraphPad Prism6 software (version 6, GraphPad software Inc., San Diego, CA, United States). Data are presented as means ± SEM. Statistical comparisons were analyzed by a Student’s *t*-test. In all cases, a value of *p* < 0.05 was considered significant.

## 3 Results

### 3.1 MN-anti-miR10b potentiates the cytotoxicity of TMZ in both U251 and LN229 cells

**FIGURE 1:**
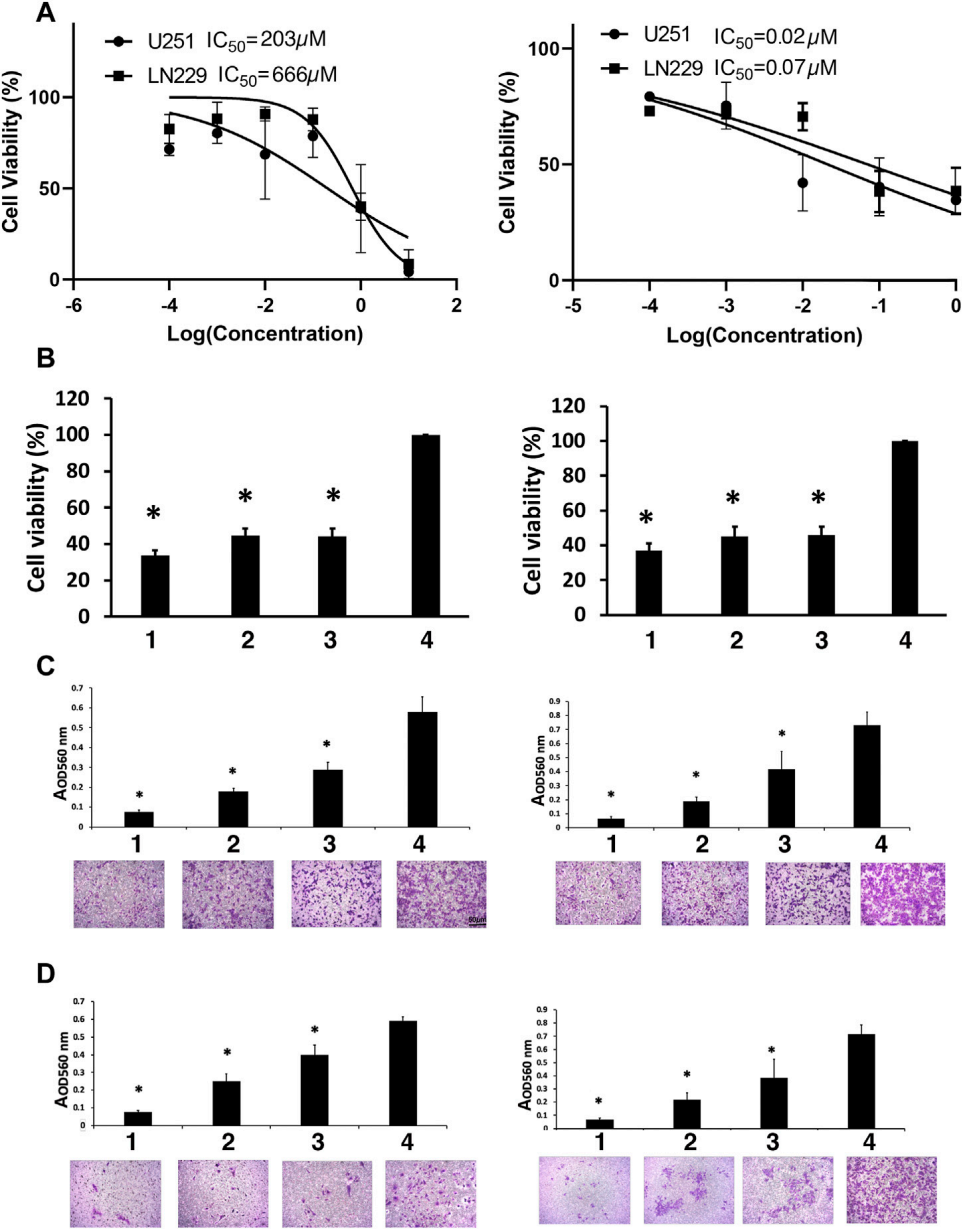
Viability studies. **(A)** Dose-response curves for TMZ (left) and MN-anti-miR10b (right). **(B)** The effect of the combination treatment of TMZ and MN-anti-miR10b used at IC50 concentrations on U251 (left) and LN229 (right) cells. TMZ and MN-anti-miR10b used as monotherapy or in combination significantly decreased the viability of U251 and LN229 cells (*p* < 0.05). Conditions as indicated: 1—TMZ + MN-anti-miR10b; 2—TMZ; 3—MN-anti-miR10b; 4—PBS. **(C)** Migration of U251 (left) and LN229 (right) cells treated with IC50 concentrations of TMZ, MN-anti-miR10b or their combination was significantly reduced (*p* < 0.05). Cells treated with PBS served as control. Conditions as indicated: 1—TMZ + MN-anti-miR10b; 2—TMZ; 3—MN-anti-miR10b; 4—PBS. **(D)** Invasion of U251 (left) and LN229 (right) cells treated with IC50 concentrations of TMZ, MN-anti-miR10b or their combination was significantly reduced (*p* < 0.05). Cells treated with PBS served as control. Conditions as indicated: 1—TMZ + MN-anti-miR10b; 2—TMZ; 3—MN-anti-miR10b; 4—PBS. Scale bar = 50 µm for **(C,D)**.

In order to establish if MN-anti-miR10b affects the viability of GBM cells and if its effects can amplify the cytotoxicity of TMZ, we first constructed dose-response curves for TMZ and MN-anti-miR10b in U251 and LN229 cells (Figure 1A) after confirming accumulation of MN-anti-miR10b in both cell lines by fluorescence microscopy (Supplementary Figure S2). Based on the IC50 concentrations calculated from the dose-response curves, we evaluated the cytotoxicity of TMZ and MN-anti-miR10b alone or in combination in both cell lines. The results indicated that both TMZ and MN-anti-miR10b when used as monotherapy decreased the viability of U251 and LN229 cells. Combination treatment with MN-anti-miR10b and TMZ showed increased cytotoxicity in both cell lines, which was significant compared to untreated controls (Figure 1B).

Quantitatively, in U251 cells, the combination treatment reduced cell viability to 33.7% ± 2.7% compared to the untreated controls (*p* < 0.05). MN-anti-miR10b alone reduced viability to 44.3% ± 4.3% of the cells while TMZ monotherapy resulted in 44.2% ± 4.0% compared to untreated controls. In LN229 cells, the combination treatment reduced cell viability to 37.2% ± 3.8% compared to 46.5% ± 4.7% for MN-anti-miR10b and 45.1% ± 5.7% for TMZ as monotherapies (Figure 1B). Incubation with unconjugated iron oxide nanoparticles or nanoparticles conjugated to scrambled oligonucleotides in increasing concentrations showed no reduction of cell viability in any cell line (Supplementary Figure S3).

Clearly, treatment alone or in combination cause decrease in cell viability compared to untreated cells. However, the differences in cell viability between the cells treated with either TMZ or MN-anti- miR10b alone or with the combination of the two were not significant suggesting that there were no additive or synergistic effects when the two drugs were co-administered at this dose regimen.

### 3.2 MN-anti-miR10b reduces the migration and invasion capabilities of GBM cells

To investigate whether inhibition of miR-10b by MN-anti-miR10b alone or in combination with TMZ could inhibit glioma cell migration, a transwell migration assay was performed. We treated U251 and LN229 cells with IC50 concentrations of TMZ, MN-anti-miR10b or their combination, while PBS-treated cells served as control. We showed that treatment with MN-anti-miR10b resulted in a 49.5% ± 10.7% reduction in the migration of U251 cells relative to PBS treated cells (*p* < 0.05). Treatment with TMZ resulted in a 68.7% ± 1.9% reduction in migration (*p* < 0.05). The combination treatment was highly effective and resulted in a 87.1% ± 1.9% inhibition of tumor cell migration compared to control cells (*p* < 0.05, Figure 1C). In the LN229 cell line, treatment with MN-anti-miR10b alone, TMZ alone, and the combination of the two resulted in a 43.6% ± 12.8% (*p* < 0.05), 73.6% ± 7.2% (*p* < 0.05), and 90.7% ± 2.7% (*p* < 0.05) inhibition of tumor cell migration compared to control cells, respectively (Figure 1C).

To measure the effect of MN-anti-miR10b alone or in combination with TMZ on glioma cell invasiveness, we employed a transwell invasion assay. The number of invasive cells in cultures treated with MN-anti-miR10b alone, TMZ alone, and the combination of the two was significantly reduced relative to controls for both cell lines (Figure 1D) with the combination treatment producing the highest effect—85.9% ± 4.2% in U251 cells (*p* < 0.05, Figure 1D, left) and 88.9% ± 2.4% in LN229 cells compared to corresponding untreated cells (*p* < 0.05, Figure 1D, right).

Most importantly, both migration and invasion in these cell lines were significantly reduced compared to each monotherapy (*p* < 0.05).

The results of the viability experiment (Figure 1B) showed that a significant number of cells (between 63% and 66%) lost their viability leaving only 33.7% ± 2.7% (U251) and 37.2% ± 3.8% (LN229) of cells viable after the combination treatment. However, even higher effect was exerted by this treatment on cell migration and invasion (87.1% ± 1.9% and 85.9% ± 4.2% for U251% and 90.7% ± 2.7% and 88.9% ± 2.4% for LN229 respectively). These results indicate that even though a portion of cells remained viable after MN-anti-miR10b + TMZ treatment, they lost their ability to migrate and invade.

**FIGURE 2:**
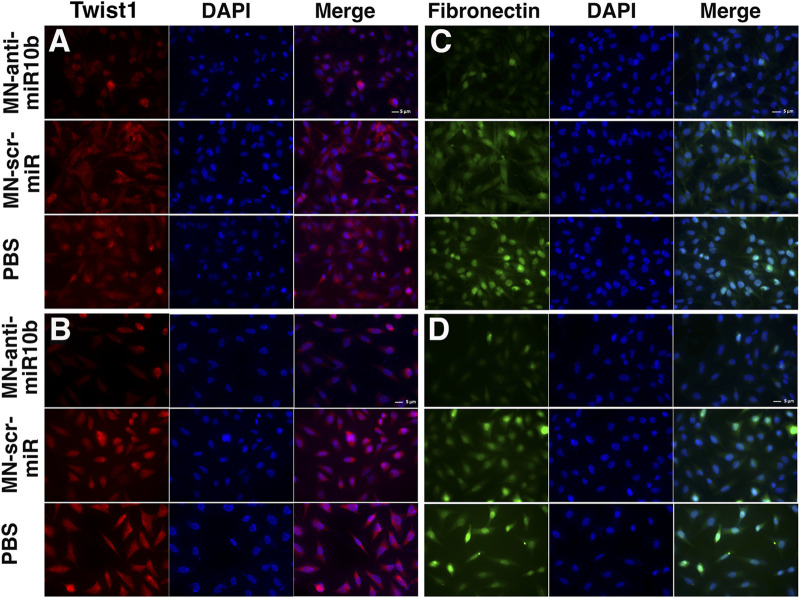
Fluorescence microscopy of the U251 **(A,B)** and LN229 **(C,D)** cells treated with MN-anti-miR10b, Mn-scr-miR and PBS and stained for Twist1 **(A,C)** and Fibronectin **(B,D)**. Scale bar = 5 µm. Quantitation is shown in Supplementary Figure S4.

Although of non-epithelial origin, Epithelial-to-Mesenchymal Transition (EMT) features and mechanisms have been demonstrated in GBM (Kubelt et al., 2015) and are associated with increased characteristic infiltration into the brain parenchyma, as well as with extreme resistance to conventional treatments (Mentlein et al., 2012; Bhat et al., 2013; Mehta and Lo Cascio, 2018). In order to investigate how MN-anti-miR10b affects EMT status of GBM cells, we analyzed the expression of two current common markers of EMT, Twist1 and Fibronectin [both upregulated in EMT (Kubelt et al., 2015)] subjected to treatments with MN-anti-miR10b, MN-scr-miR and PBS as control. As shown in Figure 2, both LN229 and U251 cells demonstrated high expression of these two markers in MN-scr-miR and PBS treated cells. In contrast, MN-anti-miR10b treated cells showed a drastic decrease in Twist1 and Fibronectin expression levels (quantitation is shown in Supplementary Figure S4). These cells also obtained a less mesenchymal morphology, as they became round and of cobblestone-appearance. These results indicated that MN-anti-miR10b inhibited the expression of EMT markers in agreement with the results of the migration and invasion analysis showing that the latter capabilities of GBM cells were weakened by the therapeutic.

### 3.3 MN-anti-miR10b and TMZ have opposing effects on miR-10b expression

**FIGURE 3:**
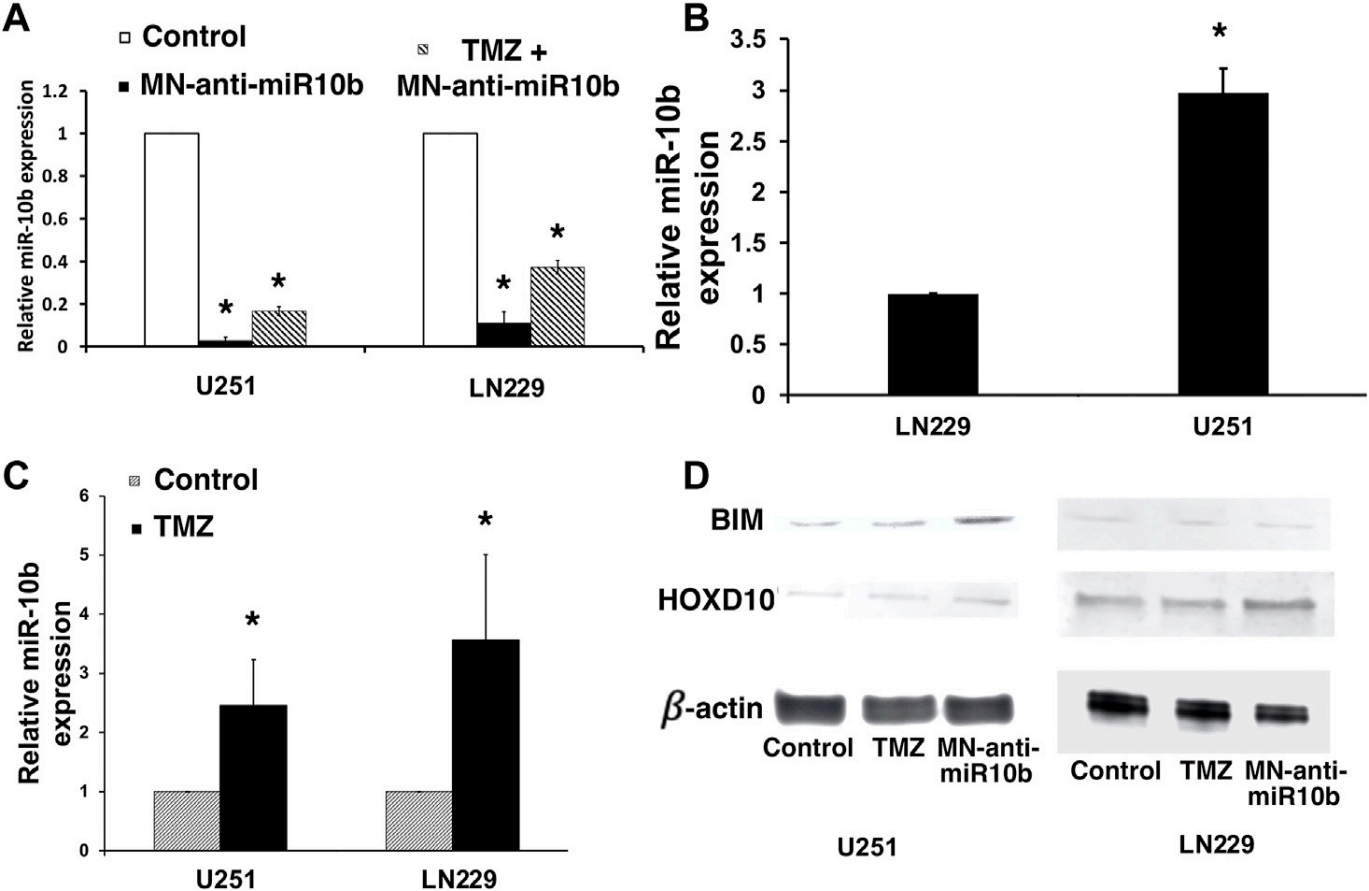
Studies of miR-10b expression in treated cells. **(A)** miR-10b expression in U251 and LN229 cells was significantly reduced after treatment with MN-anti-miR10b (*p* < 0.05, open bar vs. black bar). However, combination treatment (striped bars) resulted in higher miR-10b expression compared to monotherapy. **(B)** miR-10b expression in untreated U251 and LN229 cells. **(C)** miR-10b expression in U251 and LN229 cells was increased after treatment with TMZ (*p* < 0.05). **(D)** Western blot analysis: changes in HOXD10 and BIM expressions after treatment with MN-anti-miR10b or TMZ (left—U251; right—LN229). Quantitative data are shown in Supplementary Figure S7.

To determine if in combination with TMZ, treatment with MN-anti-miR10b retains its capacity to inhibit the miR-10b target, we performed qRT-PCR to measure miR-10b expression in cells treated with MN-anti-miR10b alone, TMZ alone or in combination. Similar to our findings in metastatic breast cancer cells (Yigit et al., 2013), MN-anti-miR10b inhibited miR-10b in both GBM cell lines. Specifically, MN-anti-miR10b decreased miRNA-10b expression in U251 cells by 50-fold, and in LN229 cells by 10-fold (*p* < 0.05, Figure 3A, open bars vs. black bars). This difference is expected since miR-10b expression in U251 cells, which are PTEN mutant, is significantly higher than in PTEN-wild type LN229 (Figure 3B, *p* < 0.05). The differential miR-10b expression between the two cell lines is also supported by previous studies (Gabriely et al., 2011). Unexpectedly, we found that treatment with TMZ alone at IC50 increased miRNA-10b expression by 2.5-fold in U251 cell line and 3.5-fold in LN229 cells (Figure 3C, *p* < 0.05). Similarly, we observed the same result not only in human cells, but also in murine GL261 cells. Likewise, miR-10b expression in these cells was decreased after treatment with MN-anti-miR10 (Supplementary Figure S5). Interestingly, T98G cells expressing high level of O^6^-methylguanine DNA-methyltransferase (MGMT), the main regulator of TMZ resistance in glioblastomas also showed increase in miR-10b expression after incubation with TMZ similar to all other cell lines tested and its decrease after treatment with MN-anti-miR10b (Supplementary Figure S6A). As expected, treatment with MN-anti-miR10 decreased miR-10b expression while MN-scr-miR or PBS left it unchanged. At the same time, MGMT expression in these cells remained unchanged following either treatment (Supplementary Figure S6B). These data point to the fact that the phenomenon of miR-10b upregulation after TMZ treatment has a universal effect on GMB cells regardless of their MGMT status. Consequentially, MN-anti-miR10b and TMZ demonstrated opposing effects on the expression of miR-10b targets which we examined in order to gain mechanistic understanding of the observed phenomena. We performed Western blotting of HOXD10 and BIM, miR-10b targets identified earlier (Gabriely et al., 2011; Teplyuk et al., 2015; Yoo et al., 2015) in U251 and LN229 cells. As shown in Figure 3D, the expression of HOXD10 and BIM was clearly suppressed after TMZ treatment in both cell lines. This was consistent with the qRT-PCR results showing that TMZ increased miR-10b expression (Figure 3C, *p* < 0.05). We also confirmed that treatment with MN-anti-miR10b increased HOXD10 and BIM protein expression in both cell lines (Figure 3D) in agreement with our previous findings (Yoo et al., 2015). Quantitative data for Western blot analysis are shown in Supplementary Figure S7.

This finding could explain the absence of additivity or synergism when TMZ and MN-anti-miR10b were combined. In particular, the induction of miR-10b by TMZ could serve as an adaptive response protecting the tumor cells from undergoing apoptosis in response to a cytotoxic stimulus. That way, in this experimental setting, induced miR-10b expression by TMZ protected cells from death effectively negating the action of MN-anti-miR10b. This effect was obvious in U251 and LN229 cells treated with TMZ and MN-anti-miR10 combination (Figure 3A, striped bars). Here, miR-10b expression was higher due to TMZ induction compared to MN-anti-miR10b monotherapy.

These findings prompted us to explore different combination treatment regimens at different concentrations of the drugs as described below.

### 3.4 Optimization of the combination treatment with MN-anti-miR10b and TMZ results in effective induction of tumor cell death

**FIGURE 4:**
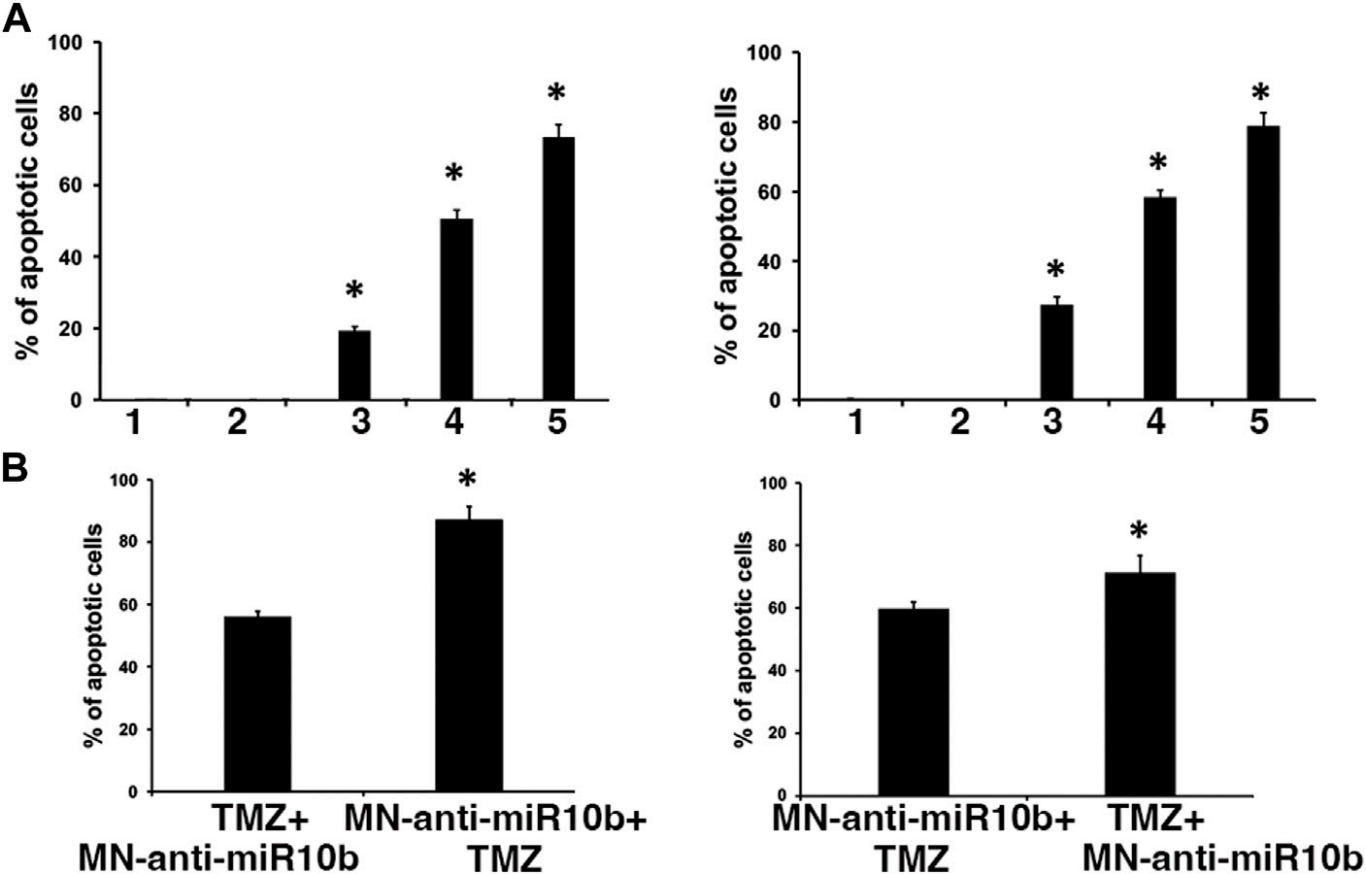
Combination treatment studies. **(A)** Simultaneous treatment of U251 (left) and LN229 (right) cells with MN-anti-miR10b and TMZ at IC90 and IC15 respectively resulted in significant induction of apoptosis (*p* < 0.05) Conditions as indicated: 1—PBS; 2—MN-scr-miR; 3—MN-anti-miR10b; 4—TMZ; 5—TMZ + MN-anti-miR10b. **(B)** Sequential treatment of U251 (left) and LN229 (right) cells with MN-anti-miR10b and TMZ. Incubation with MN-anti-miR10b followed by incubation with TMZ led to a significantly higher induction of apoptosis compared to the regimen in which TMZ was added first in both cell lines (*p* < 0.05).

Having observed that IC50 doses of TMZ increased miR-10b expression in glioma cells, we performed combination treatment studies with two major goals in mind. First, we wanted to test whether sub-therapeutic doses of TMZ would result in a more significant induction of cytotoxicity in combination with MN-anti-miR10b. To test this, we treated cells by co-administering MN-anti-miR10b at increased, IC90 dose and at significantly reduced (IC15) dose of TMZ. While monotherapies with MN-anti-miR10b and TMZ resulted in induction of apoptosis in both cells lines, the combination treatment produced significantly higher levels of apoptosis (73.4% ± 3.4% for U251% and 78.9% ± 3.8% LN229, *p* < 0.05, Figure 4A). When the inactive control, MN-scr-miR was used instead of MN-anti-miR10b, there were no significant effects in either cell line.

Secondly, we tested different sequential treatment regimens to seek the most effective one. Cells were treated either with IC15 dose of TMZ followed by MN-anti-miR10b 24 h later, or with MN-anti-miR10b followed by IC15 dose of TMZ 24 h later. As shown in Figure 4B, incubation with MN-anti-miR10b followed by incubation with TMZ led to a significantly higher induction of apoptosis compared to the regimen in which TMZ was added first (56% ± 1.7% vs. 87.1% ± 4.25% for U251% and 59.9% ± 2% vs. 71.4% ± 4% for LN229, *p* < 0.05).

**FIGURE 5:**
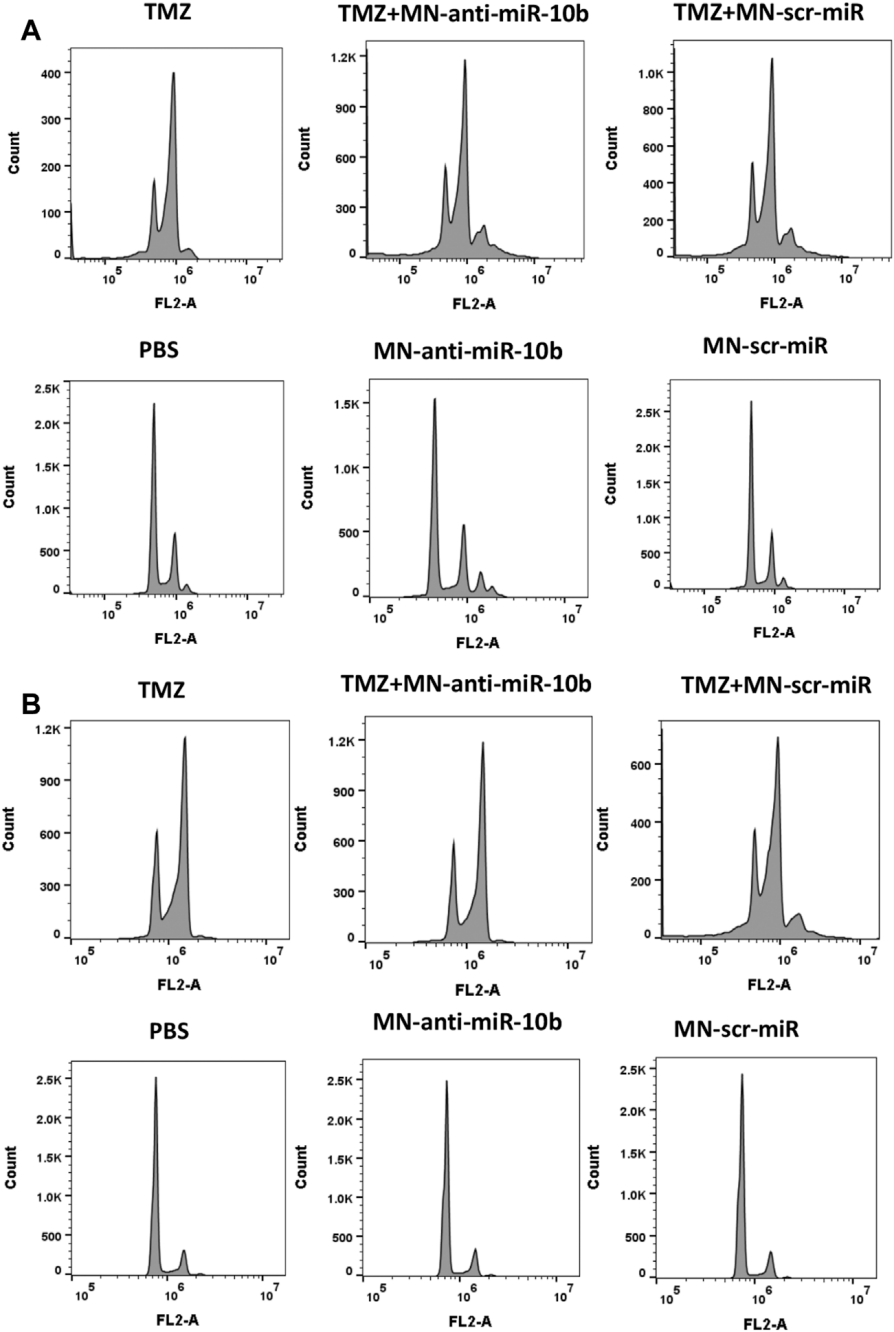
Cell cycle analysis revealed that inhibition of the cell cycle was induced by TMZ while MN-anti-miR10b apoptotic effect was cell-cycle independent in both U251 **(A)** and LN229 **(B)** cells. Quantitation is shown in Supplementary Table S2.

To gain further insight into why a subtherapeutic dose of TMZ was so effective when combined with MN-anti-miR10b, we analyzed the effect of the combination treatment on the cell cycle of the two GBM cell lines. This study revealed that TMZ tends to induce polyploidy and G2/M arrest in both U251 and LN229 cells (Figures 5A, B; quantitative analysis is shown in Supplementary Table S2), which was in accordance with the previously reported data (Zhang S. et al., 2012). Importantly, cells treated with TMZ + MN-scr-miR and TMZ + MN- anti-miR10b show the same pattern as TMZ alone, while PBS, MN-anti-miR10b and MN-scr-miR showed no effect on the cell cycle. Clearly, the inhibition of the cell cycle appeared to extend solely from the TMZ in either mono- or combination treatment, and the observed apoptotic effect of MN-anti-miR10b was cell-cycle independent. These results are consistent with the results from our previous combination treatment studies in breast cancer and suggest that inhibition of the cell cycle by TMZ may potentiate the pro-apoptotic effect of MN-anti-miR10b (Yoo et al., 2015).

## 4 Discussion

The recent discovery of the relevance of regulatory miRNAs to human cancer led to dramatic changes in our knowledge of cancer pathophysiology (Rolle, 2015). Dysregulated miRNA expression is frequently found in different human cancers including glioblastoma. One of them, miR-10b, is highly expressed in all GBM subtypes, while absent in normal neuroglial cells of the brain (Teplyuk et al., 2016). miR-10b has been implicated in cancer progression (Gabriely et al., 2011) and its inhibition has led to target derepression, and attenuated growth and progression of established intracranial GBM (Teplyuk et al., 2015; Teplyuk et al., 2016). However, the downstream effects of miR-10b inhibition have not been fully investigated. Furthermore, *in vivo* studies targeting miR-10b with antisense oligonucleotides have produced modest therapeutic results as of today (Ananta et al., 2016; Esposito et al., 2016; Teplyuk et al., 2016; Sun et al., 2019).

The combination of two or more therapeutic treatments to specifically target various aspects of carcinogenesis is a cornerstone of cancer therapy (Yap et al., 2013; Bayat Mokhtari et al., 2017). In the treatment of glioblastoma, temozolomide is used as a frontline therapy in combination with radiotherapy. Several ongoing clinical trials are testing the combination of TMZ and other chemotherapeutics such as lomustine [(Herrlinger et al., 2019), currently in a Phase 3 clinical trial]. However, the majority of combination approaches are still at the pre-clinical stage and their potential is yet to be demonstrated [reviewed in (Ghosh et al., 2018)].

In the present report, we reasoned that inhibition of miR-10b could be combined with TMZ to potentiate the overall cytotoxic effect of therapy. During the course of our studies, we found that while the combination treatment used at IC50 concentrations of both MN-anti-miR10b and TMZ decreased cell viability compared to monotherapies, this decrease was not significant. On the other hand, migration and invasion assays demonstrated that combination treatment negatively and significantly affected both features compared to chemotherapy. Furthermore, we observed a reduced expression of the known EMT markers (Twist 1 and Fibronectin) in response to treatment. There are two implications from this study. First, combination treatment at IC50 concentrations left the portion of the cells viable [33.7% ± 2.7% of U251% and 37.2% ± 3.8% of LN229 cells (Figure 1B)]. Second, even at these unfavorable concentrations the significant portion of the cells lost their ability to migrate and invade (87.1% ± 1.9% and 85.9% ± 4.2% for U251% and 90.7% ± 2.7% and 88.9% ± 2.4% for LN229 respectively). This means that in a potential treatment approach, combining TMZ with MN-anti-miR10b would synergistically inhibit tumor progression by not only reducing tumor cell viability through the effect of either TMZ or MN-anti-miR10b but also by limiting cell motility. miR-10b is a known driver of invasion and migration of tumor cells in many cancers including glioma (Ma et al., 2007; Moriarty et al., 2010; Tian et al., 2010; Gabriely et al., 2011; Guessous et al., 2013). In glioma, it induces cell invasion by modulating MMP-14 and uPAR expression via its direct target HOXD10 (Sun et al., 2011). Inhibition of miR-10b leads to a loss of the cells’ invasive ability as has been shown in previous reports (Sun et al., 2011).

During the course of our work, we unexpectedly found that TMZ monotherapy increased miR-10b expression in treated cells. This increase was accompanied by a reduction in the expression of known miR-10b targets, including HOXD10 and BIM (Gabriely et al., 2011; Sun et al., 2011; Yigit et al., 2013). Extensive literature search found only one mentioning of a similar finding where researchers demonstrated that treatment of neurospheres derived from glioma cells with TMZ resulted in the increased expression of another oncogenic miRNA, miR-21 (Rodrigues et al., 2019). Since in studies of metastatic breast cancer we showed that miR-10b acted as master regulator of metastatic cell viability (Yigit et al., 2013; Yoo et al., 2015; Yoo et al., 2017), we reasoned that its upregulation serves as a critical adaptive response to physiological stress, which enhances both tumor cell viability and motility. In the present study, the same model is supported by our results in that TMZ treatment at IC50 concentration resulted in an adaptive upregulation of miR-10b. Consequentially, we conducted a study in which we tested a series of treatment regimens including simultaneous and sequential incubations with MN-anti-miR10b and TMZ and compared the results to monotherapy. In addition, we increased the dose of MN-anti-miR10b to IC90 and reduced the dose of TMZ to IC15 reasoning that while its cytotoxic properties would still be exerted, treatment with this reduced TMZ concentration would lead to a lesser degree of miR-10b upregulation and, hence, resistance to cell death. In addition, a lower TMZ dose was motivated by the need to reduce its systemic toxicity (Stupp et al., 2005; Dixit et al., 2012; Shi et al., 2021) in a future clinical scenario.

Interestingly, we found that at these concentrations, contemporaneous treatment with MN-anti-miR10b and TMZ produced a significantly higher apoptotic effect compared to monotherapies, in stark contrast to the studies in which IC50 concentrations were used, suggesting that at lower doses of TMZ, the upregulation of miR-10b in response to cytotoxic stress was tempered, which permitted a more robust pro-apoptotic response to miR-10b inhibition. A sequential treatment regimen with the drugs showed that the therapeutic effect was significantly higher in both cell lines when TMZ was added 24 h after addition of MN-anti-miR10b (Figure 4). This finding suggested that miR-10b inhibition by MN-anti-miR10b initiated a pro-apoptotic response that rendered the tumor cells more susceptible to TMZ treatment. On the other hand, when TMZ was added first, even at a low concentration, subsequent increase in miR-10b expression most likely dampened the pro-apoptotic effect of MN-anti-miR10b, which was not able to adequately compensate this upregulation. Nevertheless, the therapeutic effect in this case was still significant (50%–60%).

Looking closely into the mechanism of action of the combination treatment, we studied the effect of the treatment on the cell cycle. Previously, TMZ has been shown to induce G2/M cell cycle arrest by causing DNA damage in human GBM cells (Zhang S. et al., 2012). Our results similarly showed the increased cell accumulation at G2/M phase (Figure 5). However, cell cycle arrest only appeared to extend from the TMZ component of the combination treatment and not from MN-anti-miR10b, which indicated a cell-cycle independent apoptotic effect of this component. A similar conclusion about the effect of TMZ on the cell cycle was reached in another study in which glioma cells were treated with PLGA nanoparticles incorporating oligonucleotides against miR-10b and miR-21 in combination with TMZ (Ananta et al., 2016). However, in that study even at the highest TMZ concentration (500 μM), which was 5-12.5 times higher than the concentrations that we used, the induction of apoptosis was achieved in only 24% of U87 cells. In contrast, our combination treatment resulted in a 71%–87% induction of apoptosis in treated cells depending on the regimen. We concluded that downregulation of miR-10b prior to treatment with TMZ is essential for obtaining this significant effect. This optimized therapeutic regimen combines the unique effect of MN-anti-miR10b on tumor cell apoptosis and motility with the specific effect of low-dose TMZ on the cell cycle, dampens the compensatory upregulation of miR-10b by TMZ, and triggers robust inhibition of tumorigenic phenotype in GBM cells. In future *in vivo* studies, we will explore combination treatments based on these results.

Looking ahead, RNA therapeutics have shown significant potential for the treatment of various diseases. However, delivery of an active RNA molecule to the cell of interest represents a significant challenge due to several factors such as inability to cross the cell membrane (Zhao et al., 2019), short half-life/digestion by nucleases (Zhang Z. et al., 2012; Stepanov et al., 2018) and entrapment in endosomes (Paliwal et al., 2015). To overcome these issues, various viral and non-viral delivery systems have been proposed. However, most of the studies have been done *in vitro*, while *in vivo* studies have shown limited success (Dong et al., 2012; Ananta et al., 2015; Vega et al., 2016; Kucukturkmen et al., 2017; Kucukturkmen and Bozkir, 2018; Malhotra et al., 2018).

Previously, we have developed a nanoparticle delivery platform consisting of iron oxide nanoparticles conjugated to oligonucleotides of interest. In addition to serving as delivery vehicles for RNA molecules, these nanoparticles serve as magnetic resonance imaging (MRI) reporters due to their superparamagnetic properties. We demonstrated the delivery of MN-anti-miR10b to various metastatic sites, including brain metastases, and eradication of metastatic lesions in animal models of breast cancer (Yigit et al., 2013; Yoo et al., 2014a; Yoo et al., 2015; Yoo et al., 2017). While the current study describes our *in vitro* work in glioblastoma cell lines, we are well poised to bring the MN-anti-miR10b platform to the next level and perform studies *in vivo*, taking into account that we have shown accumulation of the parental nanoparticles in brain tumors after systemic injection (Moore et al., 2000). We have also validated this platform for efficient intratumoral delivery of therapeutic siRNA by magnetic nanoparticles accompanied by *in vivo* imaging in GBM (Yoo et al., 2014b). In our upcoming work these nanoparticles will be used to deliver anti-miR10b oligonucleotides to animals with brain tumors in conjunction with *in vivo* MRI. We believe that the described studies are of paramount importance as they provide functional and mechanistic insights into the synergistic action of MN-anti-miR10b and TMZ and define a regimen for future vivo studies. Importantly, given the proven role played by miR-10b in various cancers beyond glioblastoma (Ma et al., 2007; Moriarty et al., 2010; Tian et al., 2010; Guessous et al., 2013) these studies could bring the promise of RNA therapeutic to patients suffering from these devastating diseases.

## Data Availability

The original contributions presented in the study are included in the article/Supplementary Material, further inquiries can be directed to the corresponding author.
