# Supplementary material for: Co-administration of temozolomide (TMZ) and the experimental therapeutic targeting miR-10b, profoundly affects the tumorigenic phenotype of human glioblastoma cells
Source: Front Mol Biosci. 2023 Jun 15;10:1179343. doi: 10.3389/fmolb.2023.1179343 (PMC10311069; doi:10.3389/fmolb.2023.1179343)

Supplementary Material

Co-administration of Temozolomide (TMZ) and the Experimental Therapeutic Targeting miR-10b, Profoundly Affects the Tumorigenic Phenotype of Human Glioblastoma Cells

Ming Chen, Bryan Kim, Neil Robertson, Sujan Kumar Mondal, Zdravka Medarova, Anna Moore^*^

*** Correspondence:** Anna Moore: [moorea57@msu.edu](mailto:moorea57@msu.edu)

# Supplementary Tables

**Supplementary Table 1.** Characterization of MN-anti-miR10b and MN-scr-miR.

|  | Iron  concentration (µM) | #amino groups/MN | #oligos/MN | MN size (nm) | Zeta potential (mV) |
| --- | --- | --- | --- | --- | --- |
| MN-anti-miR10b | 1.6 | 120 | 14 | 25.4 | +6.5 |
| Mn-scr-miR | 1.6 | 120 | 12 | 24.7 | +6.5 |

**Supplementary Table 2.** The percentage of cell distribution at different phases of the cell cycle under different treatment conditions. Quantitation of the flow cytometry data presented in Fig. 5.

| U251 |  |  |  |  |  |  |
| --- | --- | --- | --- | --- | --- | --- |
|  | Treatment | %G1 | %S | %G2/M | %<G1 | %>G2 |
|  | PBS | 67.75±2.5 | 17.65±1.6 | 15.05±0.07 | 1.46±0.54 | 0.58±0.14 |
|  | MN-anti-miR10b | 68.5±0.42 | 20.15±1.5 | 12.05±0.64 | 1.685±0.5 | 0.17±0.04 |
|  | MN-scr-miR | 72.05±1.6 | 14±1.7 | 13.8±0.42 | 2.255±0.28 | 0.39±0.11 |
|  | TMZ | 19.85±5.2 | 39.4±0.42 | 43.05±2.8 | 0.595±0.37 | 1.45±1.3 |
|  | TMZ+MN-anti-miR10b | 19.2±0.14 | 50.15±3.6 | 28.95±3.9 | 1.615±0.12 | 0.402±0.49 |
|  | TMZ+MN-scr-miR | 18.05±0.49 | 49.85±10.4 | 29.6±9.8 | 2.075±0.04 | 0.46±0.65 |
| LN229 |  |  |  |  |  |  |
|  | Treatment | %G1 | %S | %G2/M | %<G1 | %>G2 |
|  | PBS | 80.9±0.14 | 8.515±3.7 | 9.155±3.5 | 4.345±0.95 | 0.13±0 |
|  | MN-anti-miR10b | 86.1±1.8 | 9.495±0.7 | 5.805±2.9 | 3.705±1.4 | 0.311±0.34 |
|  | MN-scr-miR | 84.25±4.3 | 8.445±0.89 | 8.715±1.69 | 3.695±2.1 | 0.325±0.23 |
|  | TMZ | 21.4±5.1 | 38.25±2.5 | 40.75±1.8 | 1.385±0.04 | 1.205±0.007 |
|  | TMZ+MN-anti-miR10b | 24.45±2.1 | 33.9±1.6 | 40.15±1.5 | 2.785±0.47 | 0.275±0.1 |
|  | TMZ+MN-scr-miR | 16.45±1.8 | 54.3±4.1 | 28±7.6 | 2±0.14 | 0.755±0.23 |

# 2. Supplementary Figures


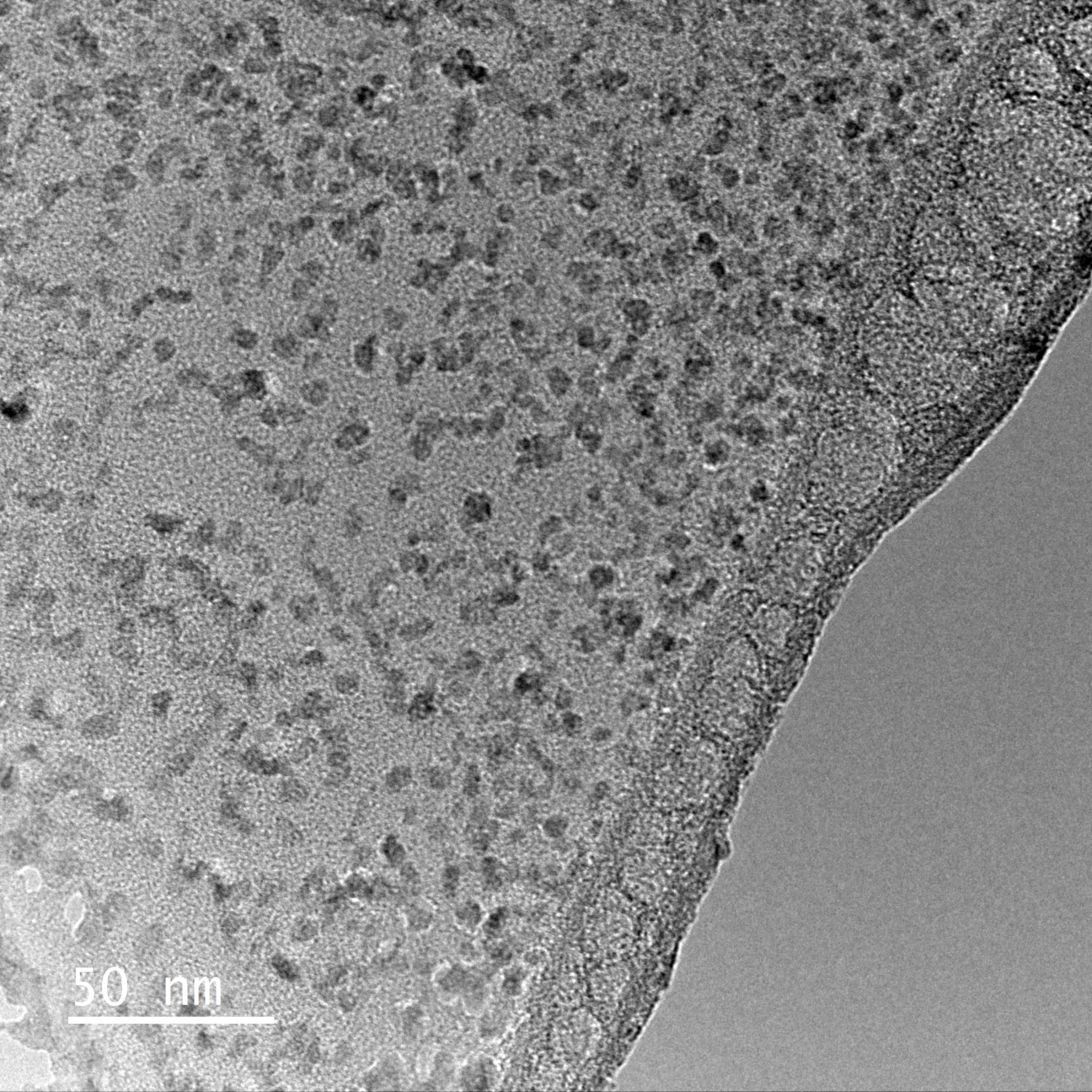


**Supplementary Figure 1.** Transmission electron microscopy (TEM) confirmed crystalline lattice structure of the nanoparticle core and its size (5.7 ± 0.5 nm).


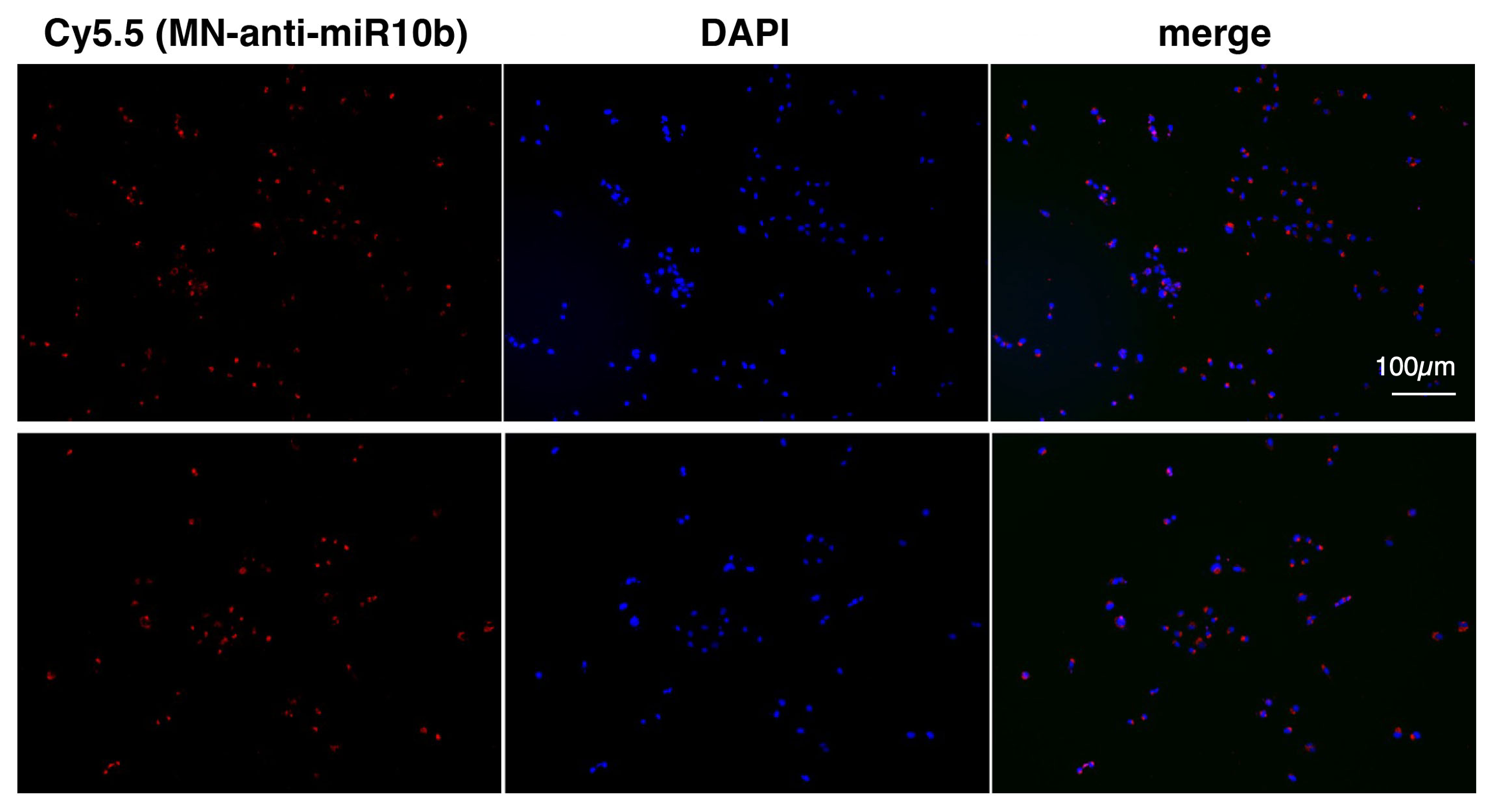


**Supplementary Figure 2.** Fluorescence microscopy demonstration accumulation of MN-anti-miR10b in U251 (top) and LN229 (bottom) cells after 2 hrs incubation.


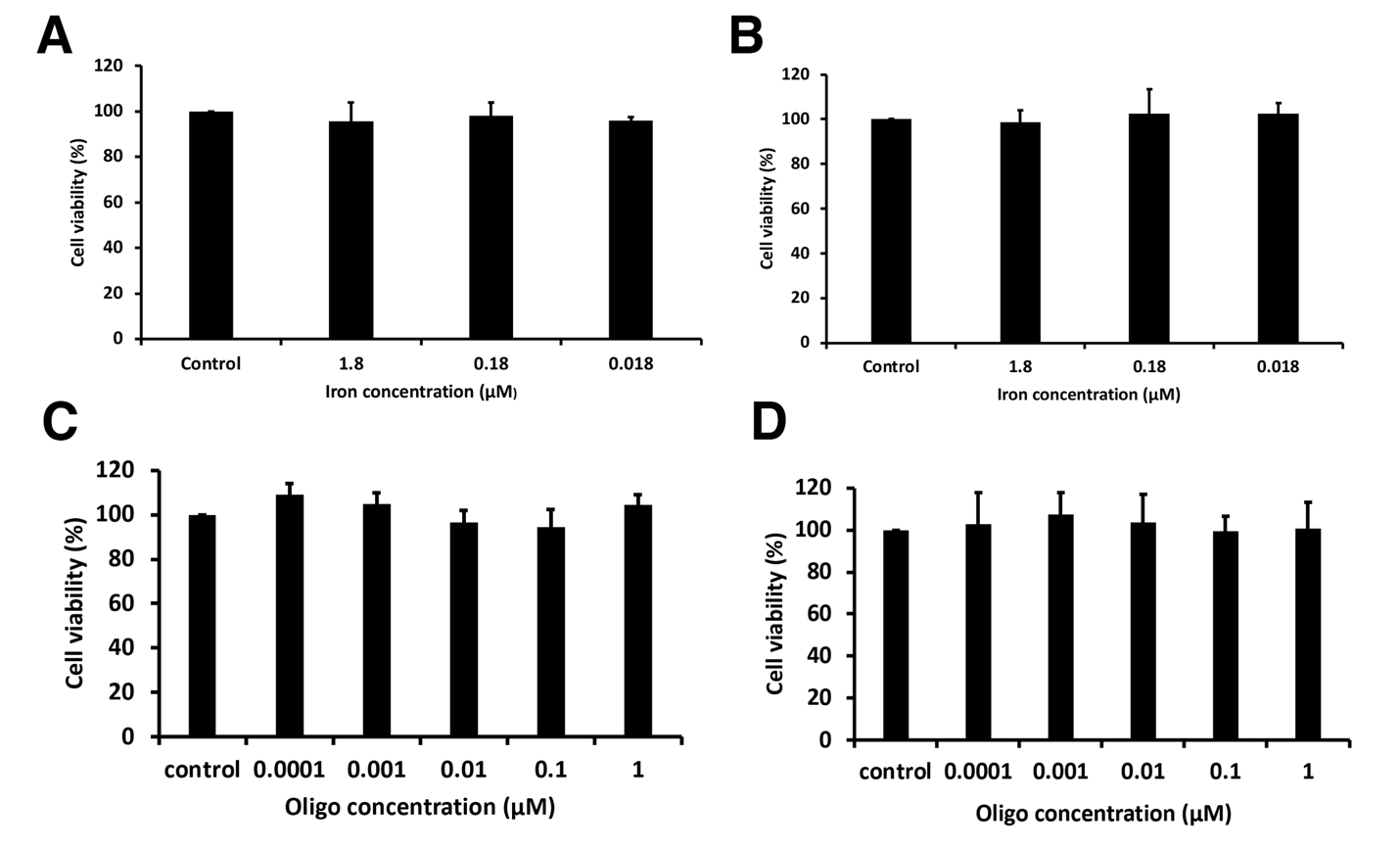


**Supplementary Figure 3.** U251 (A, C) and LN229 (B, D) cells incubated with increasing concentrations of either unconjugated nanoparticles (A, B) or scrambled control nanoparticles (MN-scr-miR) (C, D) showed no notable reduction in cell viability.

A

B

C

D

Supplementary Figure 4. Quantitative data for fluorescence microscopy of the U251 (A, B) and LN229 (C, D) cells treated with MN-anti-miR10b, Mn-scr-miR and PBS and stained for TWIST1 (A, C) and Fibronectin (B, D).


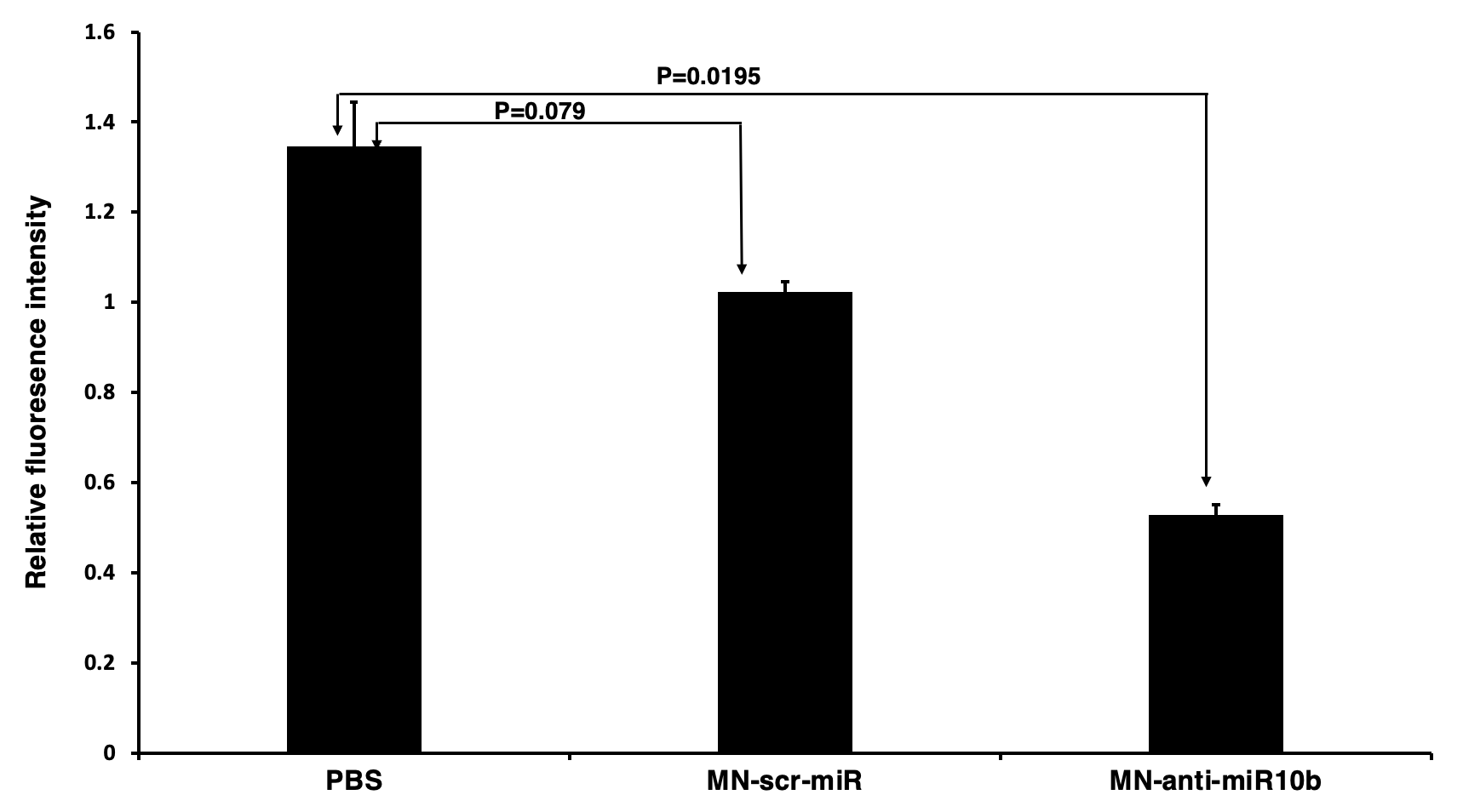


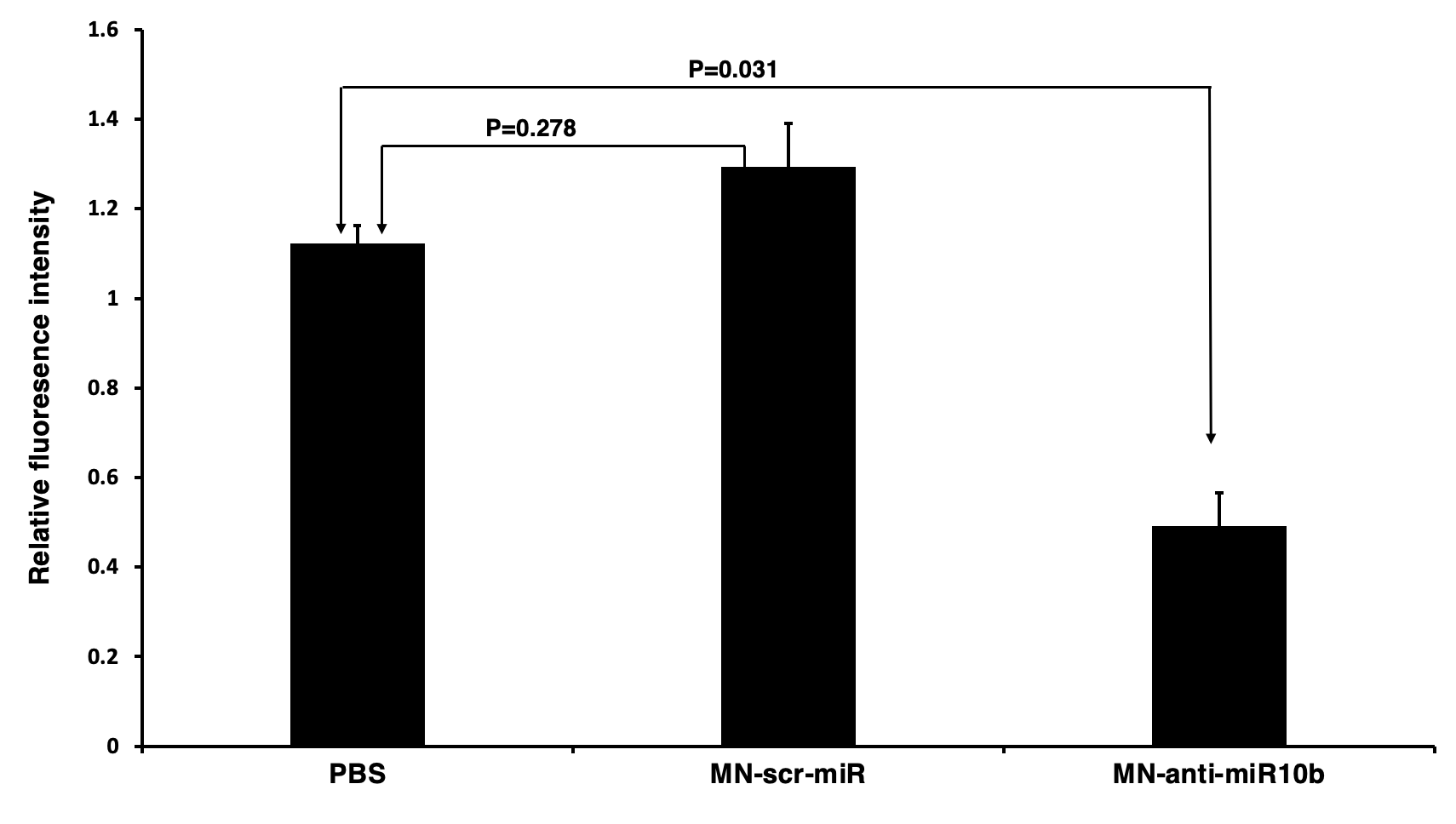


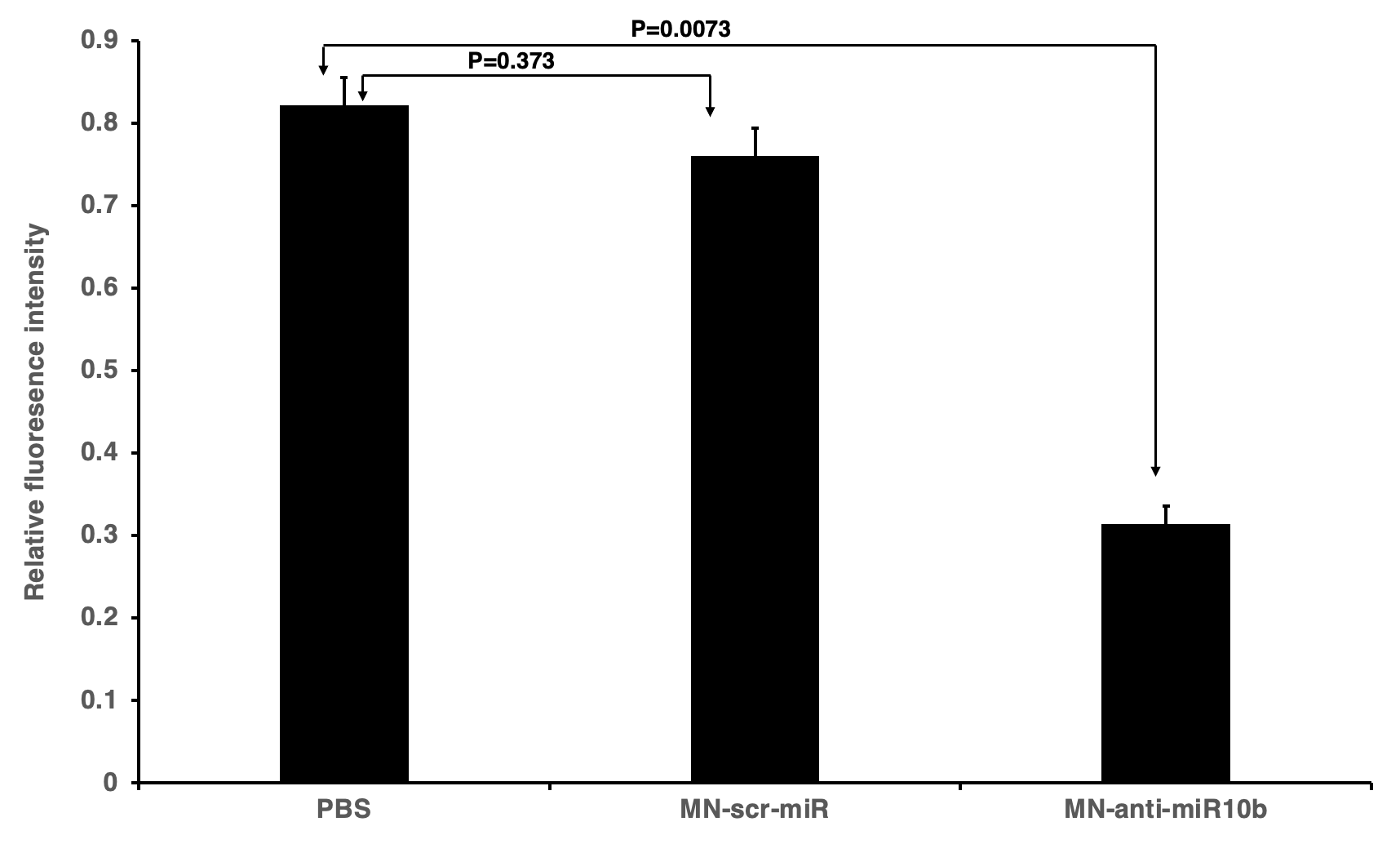


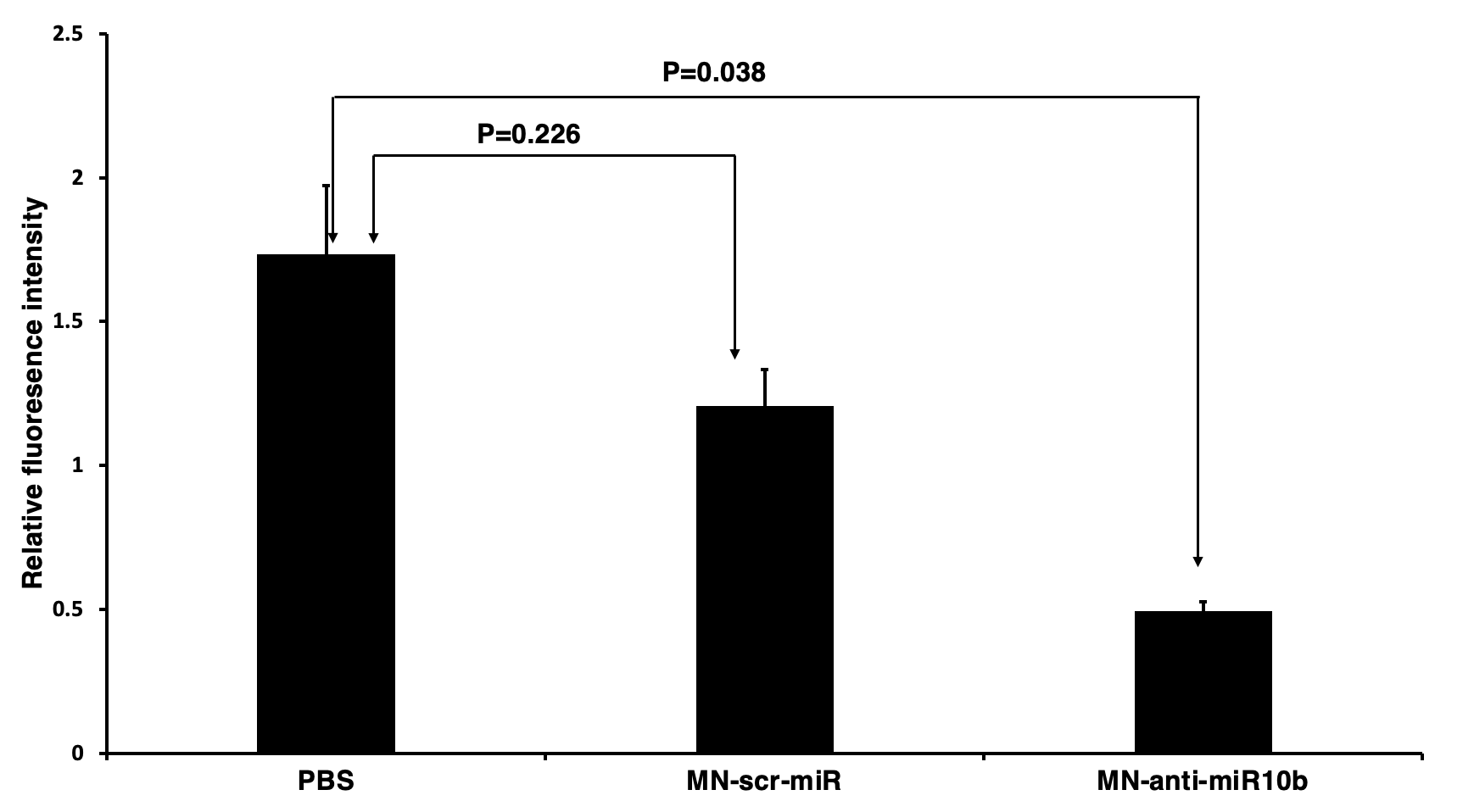


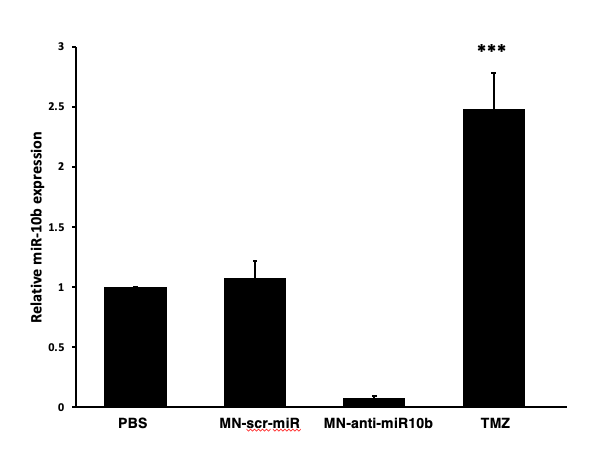


**Supplementary Figure 5.** Treatment with TMZ alone increased miRNA-10b expression in GL261 cells (p<0.001). As expected, treatment with MN-anti-miR10 decreased miR-10b expression while MN-scr-miR or PBS left it unchanged.

**A**

*

**
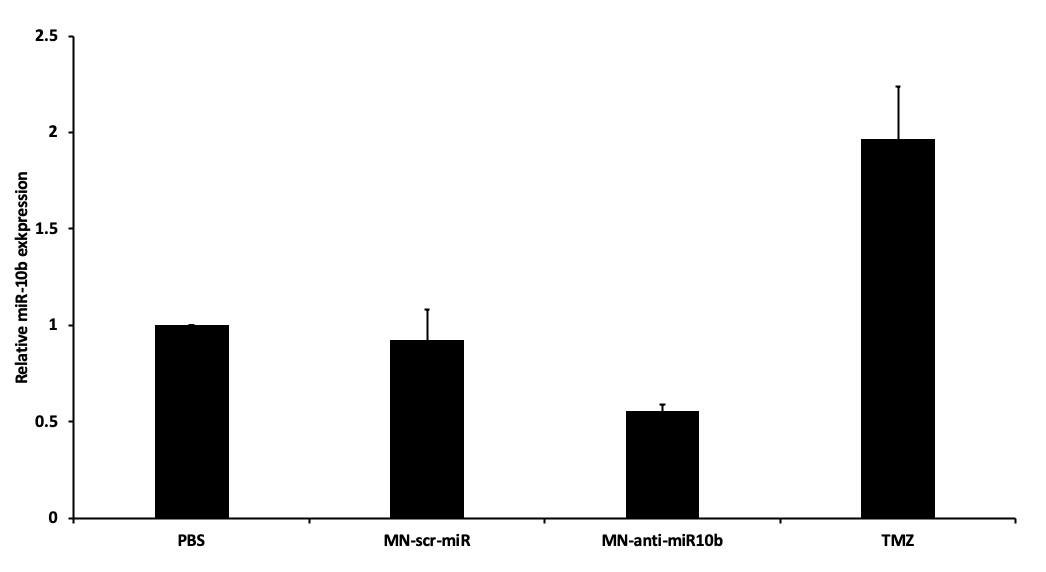
**

**B**


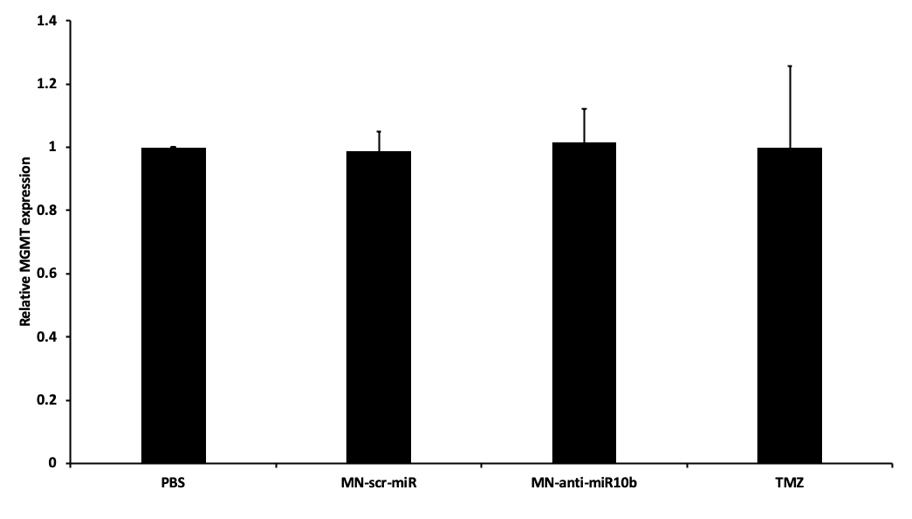


**Supplementary Figure 6.** A. Treatment with TMZ alone increased miRNA-10b expression in T98G cells (p<0.05). As expected, treatment with MN-anti-miR10 decreased miR-10b expression while MN-scr-miR or PBS left it unchanged. B. MGMT expression in T98G cells remained unchanged following either treatment.


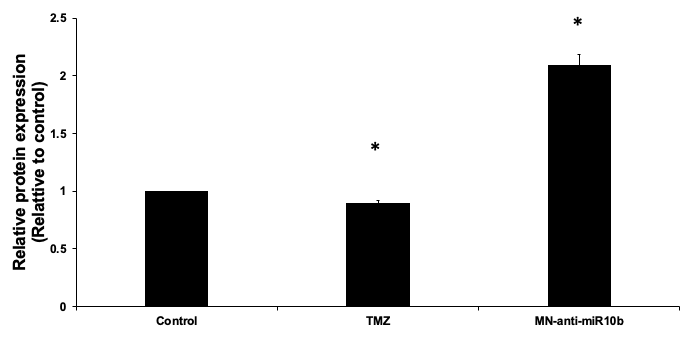

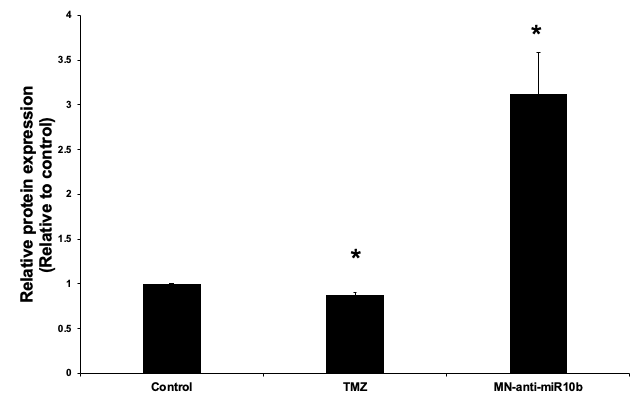

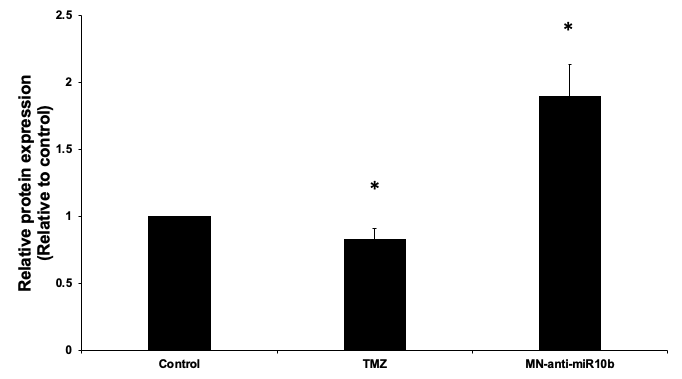
**Supplementary Figure 7.** Quantitative data for western blot analysis shown in Fig. 3D - HOXD10 expression in LN229 cells; B - HOXD10 expression in U251 cells; C – BIM expression in LN229 cells; D – BIM expression in U251 cells (p<0.05).

**D**

**C**

**B**

**A**


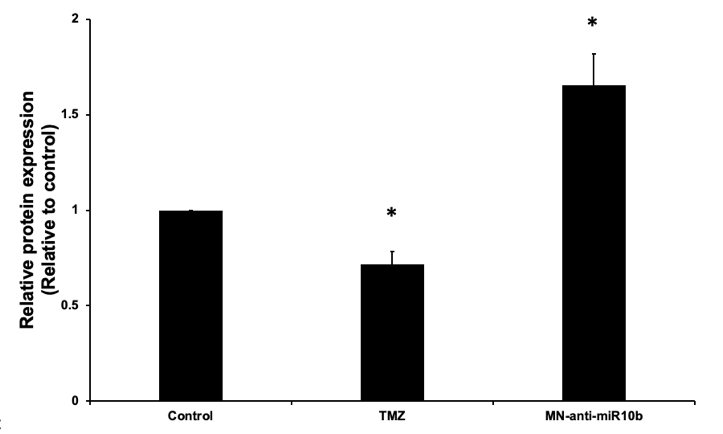

Supplement: Supplementary file 1 [file Table1.DOCX]
