# Supplementary material for: Co-administration of temozolomide (TMZ) and the experimental therapeutic targeting miR-10b, profoundly affects the tumorigenic phenotype of human glioblastoma cells
Source: Front Mol Biosci. 2023 Jun 15;10:1179343. doi: 10.3389/fmolb.2023.1179343 (PMC10311069; doi:10.3389/fmolb.2023.1179343)

Supplementary Material

Co-administration of Temozolomide (TMZ) and the Experimental Therapeutic Targeting miR-10b, Profoundly Affects the Tumorigenic Phenotype of Human Glioblastoma Cells

Ming Chen, Bryan Kim, Neil Robertson, Sujan Kumar Mondal, Zdravka Medarova, Anna Moore*

*** Correspondence:** Anna Moore: moorea57@msu.edu

1 Supplementary Tables

Supplementary Table 1. Characterization of MN-anti-miR10b and MN-scr-miR.

	Iron concentration (μM)	#amino groups/MN	#oligos/MN	MN size (nm)	Zeta potential (mV)
MN-anti-miR10b	1.6	120	14	25.4	+6.5
Mn-scr-miR	1.6	120	12	24.7	+6.5

Supplementary Table 2. The percentage of cell distribution at different phases of the cell cycle under different treatment conditions. Quantitation of flow cytometry data presented in Fig. 5.

U251						
	Treatment	%G1	%S	%G2/M	%<G1	%>G2
	PBS	67.75±2.5	17.65±1.6	15.05±0.07	1.46±0.54	0.58±0.14
	MN-anti-miR10b	68.5±0.42	20.15±1.5	12.05±0.64	1.685±0.5	0.17±0.04
	MN-scr-miR	72.05±1.6	14±1.7	13.8±0.42	2.255±0.28	0.39±0.11
	TMZ	19.85±5.2	39.4±0.42	43.05±2.8	0.595±0.37	1.45±1.3
	TMZ+MN-anti-miR10b	19.2±0.14	50.15±3.6	28.95±3.9	1.615±0.12	0.402±0.49
	TMZ+MN-scr-miR	18.05±0.49	49.85±10.4	29.6±9.8	2.075±0.04	0.46±0.65
LN229						
	Treatment	%G1	%S	%G2/M	%<G1	%>G2
	PBS	80.9±0.14	8.515±3.7	9.155±3.5	4.345±0.95	0.13±0
	MN-anti-miR10b	86.1±1.8	9.495±0.7	5.805±2.9	3.705±1.4	0.311±0.34
	MN-scr-miR	84.25±4.3	8.445±0.89	8.715±1.69	3.695±2.1	0.325±0.23
	TMZ	21.4±5.1	38.25±2.5	40.75±1.8	1.385±0.04	1.205±0.007
	TMZ+MN-anti-miR10b	24.45±2.1	33.9±1.6	40.15±1.5	2.785±0.47	0.275±0.1
	TMZ+MN-scr-miR	16.45±1.8	54.3±4.1	28±7.6	2±0.14	0.755±0.23

2. Supplementary Figures

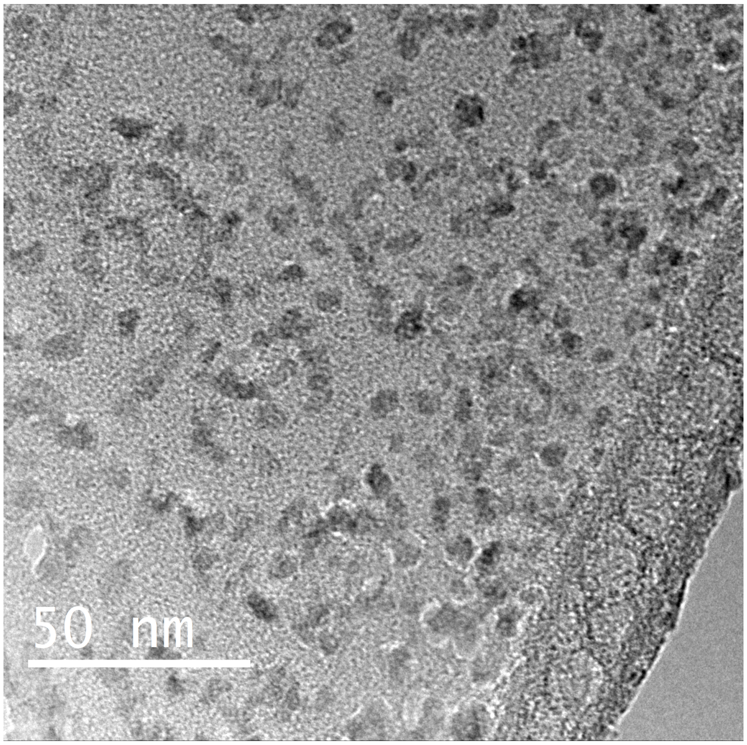

Supplementary Figure 1. Transmission electron microscopy (TEM) confirmed crystalline lattice structure of the nanoparticle core and its size (5.7 ± 0.5 nm).

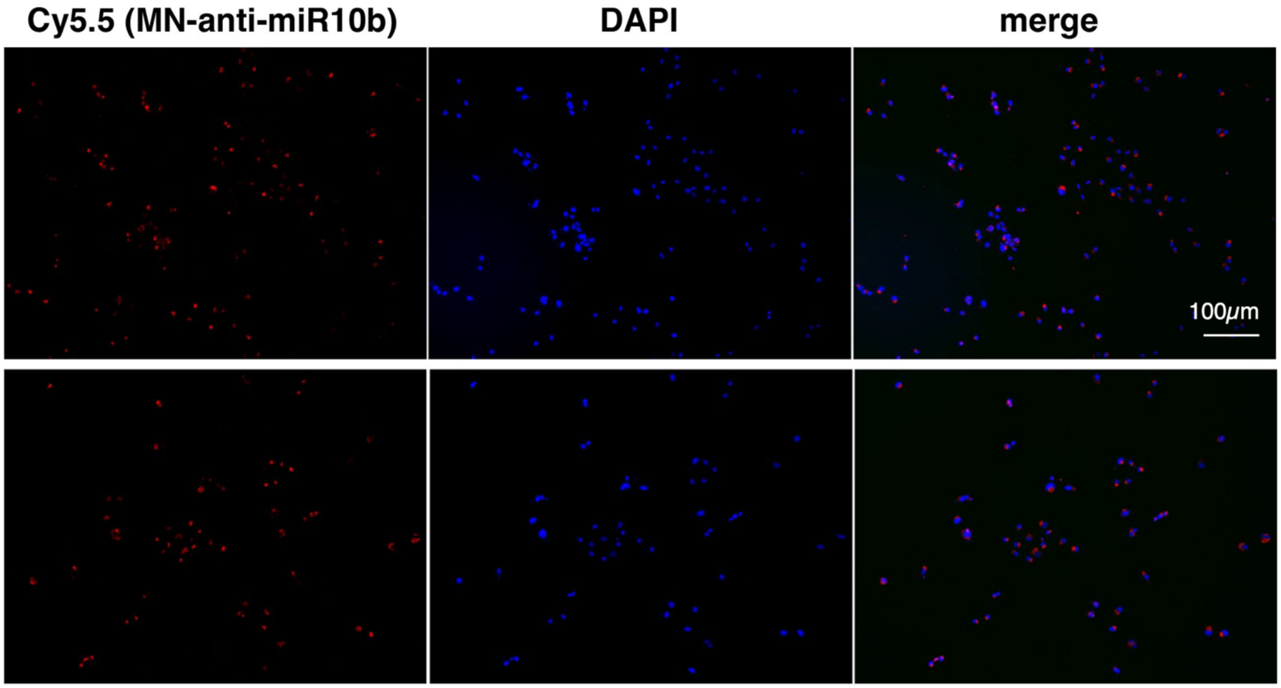

Supplementary Figure 2. Fluorescence microscopy demonstration accumulation of MN-anti-miR10b in U251 (top) and LN229 (bottom) cells after 2 hrs incubation.

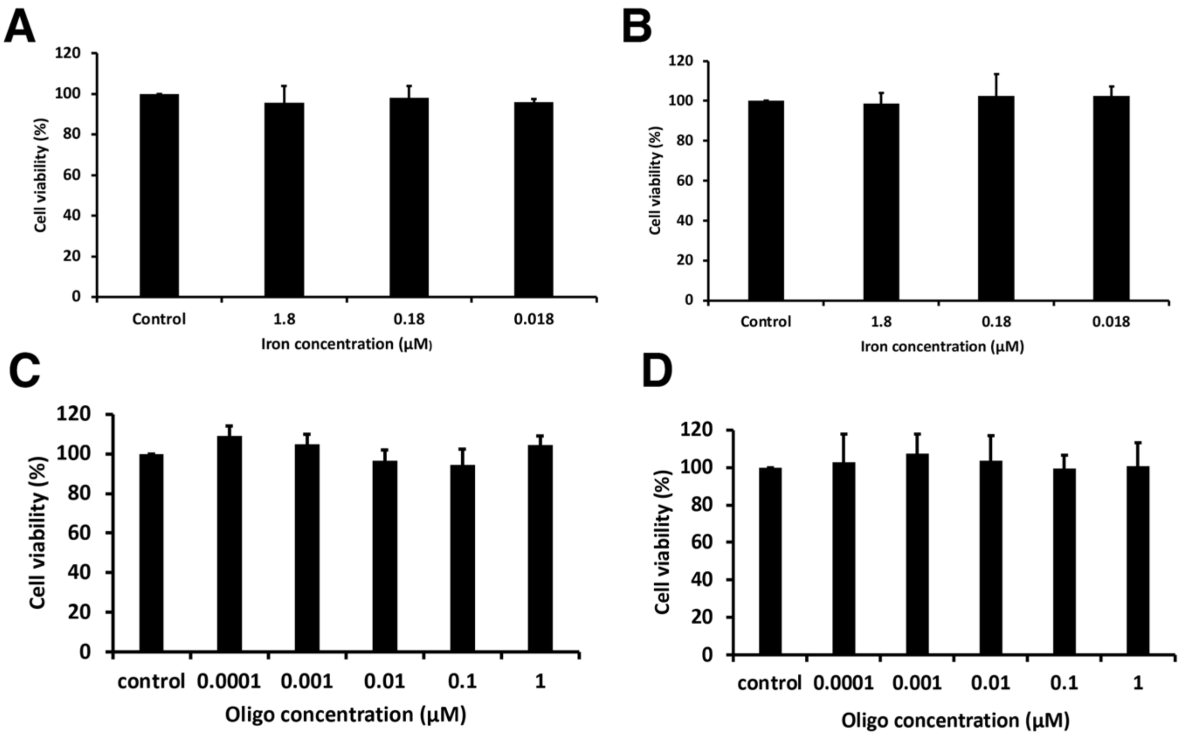

Supplementary Figure 3. U251 (A, C) and LN229 (B, D) cells incubated with increasing concentrations of either unconjugated nanoparticles (A, B) or scrambled control nanoparticles (MN-scr-miR) (C, D) showed no notable reduction in cell viability.

A

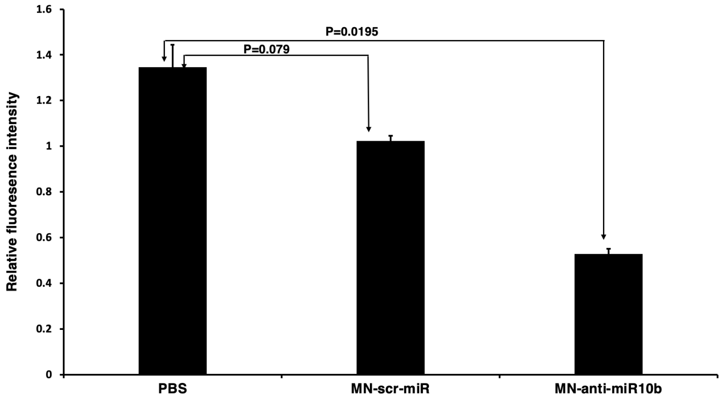

B

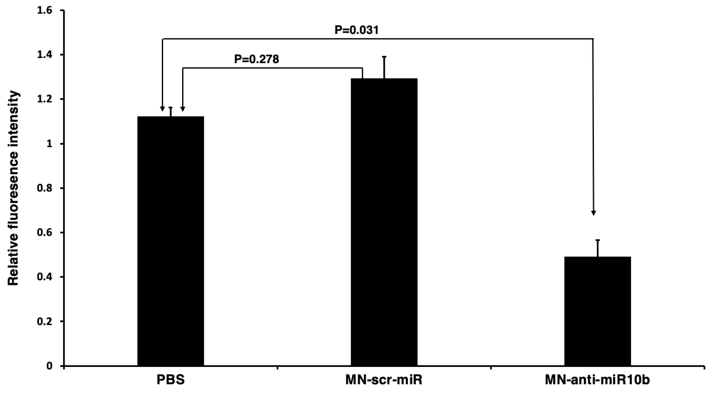

C

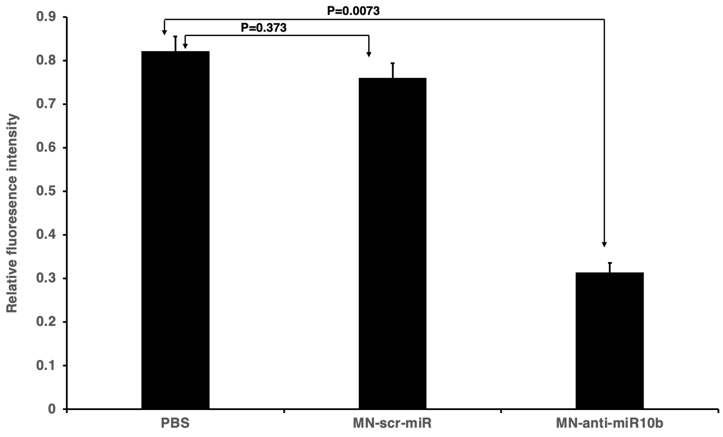

D

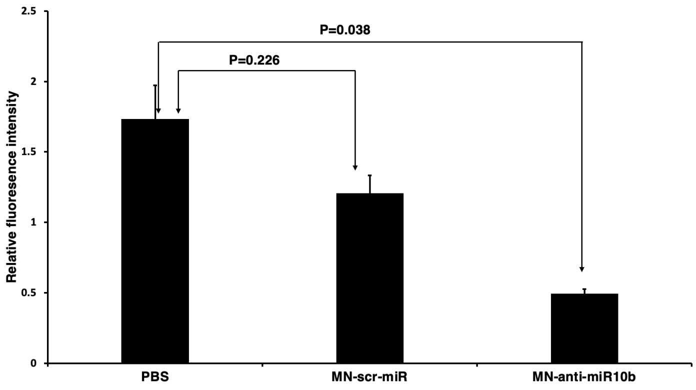

Supplementary Figure 4. Quantitative data for fluorescence microscopy of the U251 (A, B) and LN229 (C, D) cells treated with MN-anti-miR10b, Mn-scr-miR and PBS and stained for TWIST1 (A, C) and Fibronectin (B, D).

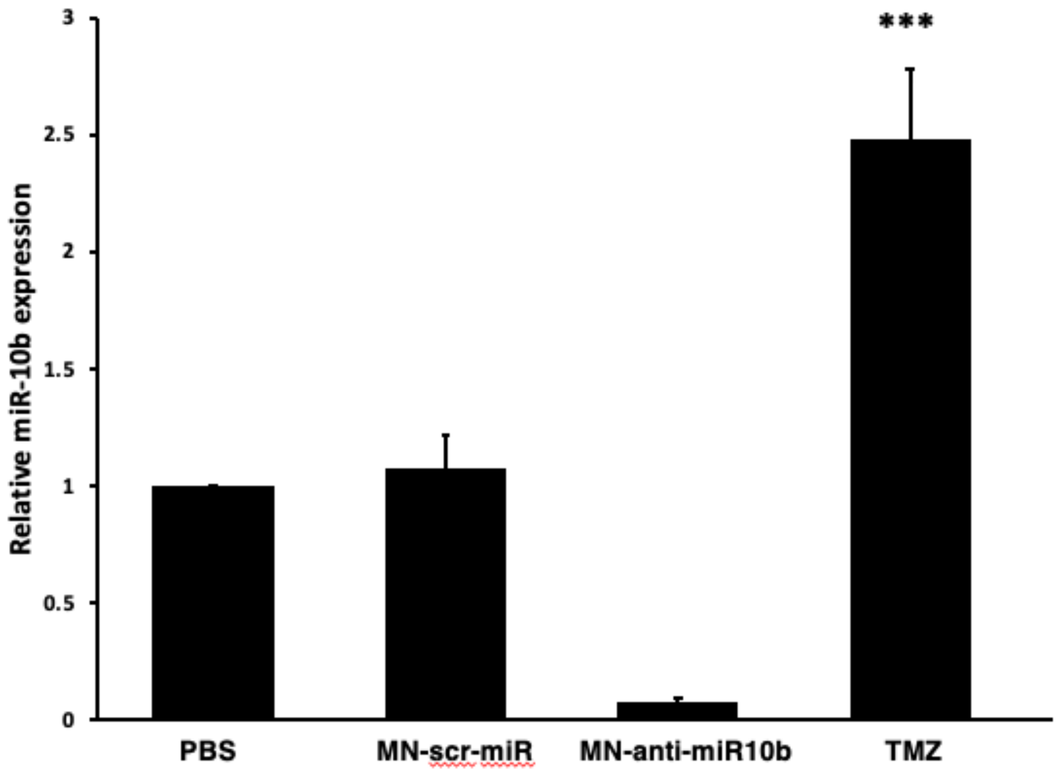

Supplementary Figure 5. Treatment with TMZ alone increased miRNA-10b expression in GL261 cells ($p<0.001$). As expected, treatment with MN-anti-miR10 decreased miR-10b expression while MN-scr-miR or PBS left it unchanged.

A

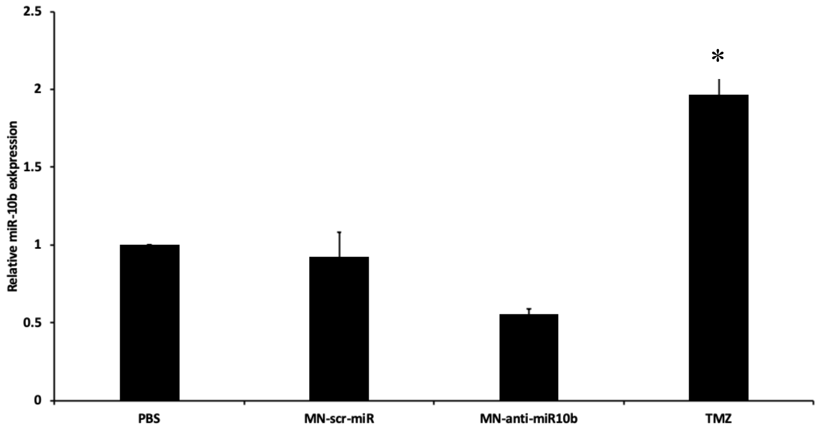

B

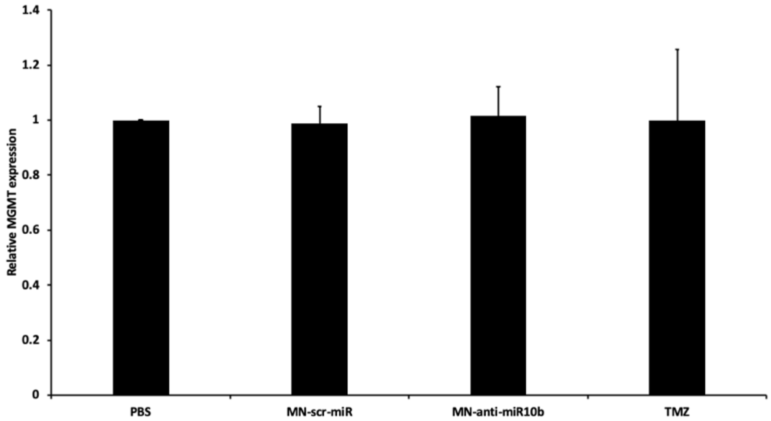

Supplementary Figure 6. A. Treatment with TMZ alone increased miRNA-10b expression in T98G cells ($p<0.05$). As expected, treatment with MN-anti-miR10 decreased miR-10b expression while MN-scr-miR or PBS left it unchanged. B. MGMT expression in T98G cells remained unchanged following either treatment.

Supplementary Figure 7. Quantitative data for western blot analysis shown in Fig. 3D - HOXD10 expression in LN229 cells; B - HOXD10 expression in U251 cells; C – BIM expression in LN229 cells; D – BIM expression in U251 cells (p<0.05).

A

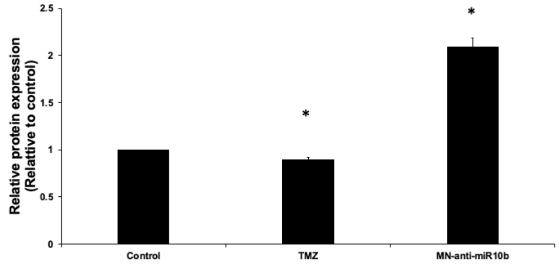

B

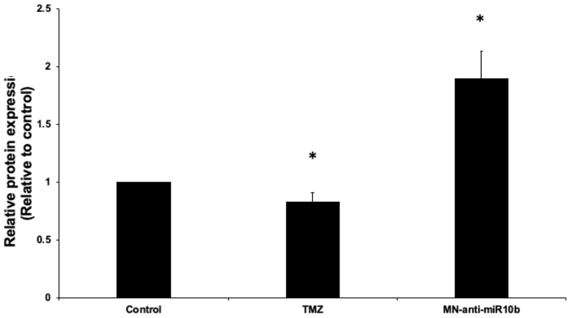

C

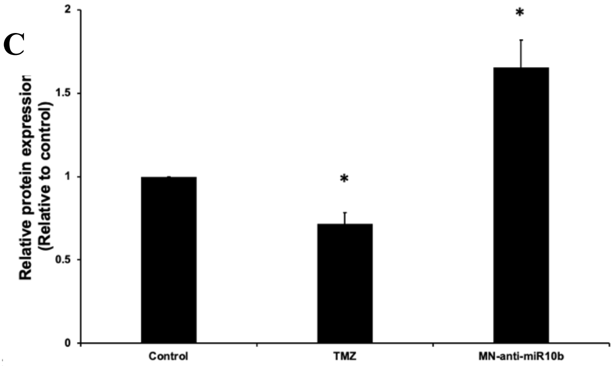

D

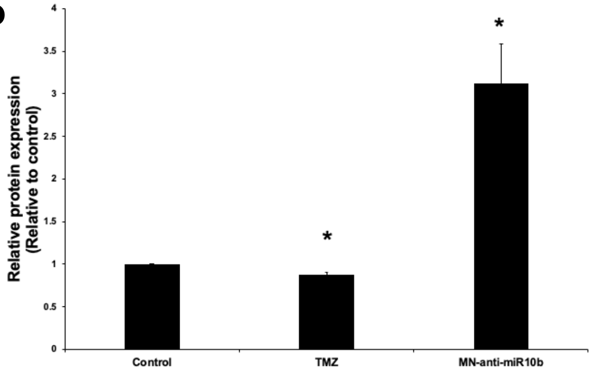

Supplement: Supplementary file 2 [file DataSheet1.PDF]
